# Preferences of people living with HIV for treatment regimen attributes: a qualitative interview study

**DOI:** 10.3389/fpubh.2026.1907747

**Published:** 2026-09-14

**Authors:** Christina Donatti, Manukiran Ravi, Selene Camargo-Correa, Ana Maria Rodriguez-Leboeuf

**Affiliations:** 1 Global Health Outcomes, ViiV Healthcare, London, United Kingdom; 2 Patient-Centered Solutions, IQVIA, Bengaluru, Karnataka, India; 3 Patient-Centered Solutions, IQVIA, Barcelona, Spain; 4 Patient-Centered Solutions, IQVIA, Madrid, Spain

**Keywords:** antiretroviral therapy, healthcare providers, HIV, long-acting injectables, personal factors, qualitative analysis, treatment attributes

## Abstract

**Introduction:**

To develop effective HIV treatments, including long-acting injectables, understanding the treatment attributes and personal factors that are significant to people living with HIV is essential.

**Methods:**

We conducted individual qualitative interviews with people living with HIV and healthcare providers (HCP) in the United States to evaluate treatment preferences if considering a hypothetical treatment switch. We performed a targeted literature review and identified five attributes related to HIV treatment: (1) administration mode, (2) administration setting, (3) administration frequency, (4) location of injection on the body, and (5) side effects. We also recorded any spontaneously mentioned attributes and other factors influencing treatment decisions. A thematic analysis was conducted using MAXQDA, with dual independent coding of 20% of transcripts and third-party reconciliation by the qualitative lead. Spanish-language transcripts were reviewed in both the original and translated versions to preserve meaning.

**Results:**

We interviewed 50 people living with HIV and 5 HCPs. Among people living with HIV, 60% were male, 62% had no prior injection treatment experience, and median (interquartile range) age was 50 (39.8–55.8) years. Side effects emerged as the highest priority attribute for people living with HIV and for HCPs, followed by administration frequency and mode. Most participants expressed a clear preference for injectable over oral administration when all injection forms (intramuscular, subcutaneous, intravenous, combinations) were considered together. Among HCP-administered injection options, 4- and 6-month intervals were widely preferred, driven by alignment with existing appointment schedules and relationships with providers.

**Conclusion:**

When considering treatment options, the highest priorities for people living with HIV are reducing side effects and administration frequency. A planned quantitative discrete choice experiment will evaluate generalizability of our results and provide evidence to support stakeholder decisions.

## 1 Introduction

Modern antiretroviral therapy (ART) regimens are highly effective for maintaining viral suppression in people living with HIV (1). However, the Centers for Disease Control and Prevention estimated that, in 2021, approximately 25% of people diagnosed with HIV in the United States did not access ART (2). Adherence to ART is affected by physical, emotional, and psychosocial challenges, which can be associated with poor health outcomes such as virologic failure and low treatment satisfaction (3). Achieving good treatment adherence requires an understanding of patient preferences (4). Thus, there is a need to evaluate the preferences of people living with HIV regarding treatment options, and to develop expanded treatment options that align with those preferences and improve the ability of individuals to access the right treatment options.

Long-acting (LA) injectable ART has the potential to address adherence challenges and improve quality of life for people living with HIV (5). Cabotegravir + rilpivirine (CAB + RPV) LA is the first complete LA injectable ART regimen approved for HIV-1 treatment (6–8). This regimen is administered by a healthcare provider (HCP) as two gluteal intramuscular injections once monthly or every 2 months (6). In phase 3 clinical trials, CAB + RPV LA demonstrated high efficacy and a good safety profile in adults with HIV who were virologically suppressed, and was non-inferior to daily oral ART (9, 10). Clinical trial participants preferred CAB + RPV LA over their previous oral regimens and reported greater treatment satisfaction (9). Furthermore, contingent on similar strong efficacy, results from discrete choice experiments (DCE), in which participants were asked to choose between hypothetical LA injectable ART versus their current oral regimen, have shown a preference for longer-acting treatment options (11, 12). In the phase 3 LATITUDE clinical trial, CAB + RPV LA demonstrated superior efficacy compared with daily oral ART in people living with HIV who have barriers to adherence, supporting its use in this population (13). Previous studies have also indicated that the potential for self-administration may be preferred by some people living with HIV (11). However, a deeper understanding of the preferences of people living with HIV regarding attributes of LA injectable ART and other treatment options is needed.

When developing new treatment options, understanding the treatment attributes considered important by people living with HIV and by HCPs, as well as personal factors that can influence their perspectives, is critical. The US Food and Drug Administration encourages medical product sponsors to collect data on patient preferences and uses this information to inform regulatory decision-making (14). Patient preference information is also of interest to health technology assessment (HTA) bodies and may impact payer decision-making (15). While previous DCEs evaluated the preferences of people living with HIV regarding limited hypothetical LA injectable options, the full range of patient preferences regarding HIV treatment attributes and the factors influencing these preferences remain poorly understood. This qualitative interview study aimed to evaluate the preferences of people living with HIV and of HCPs in the United States regarding attributes associated with different HIV treatment options, including a range of potential future LA injectable profiles, and the factors influencing these preferences.

## 2 Methods

### 2.1 Study design

This was a cross-sectional, non-interventional study based on semi-structured, individual qualitative interviews with both people living with HIV and HCPs in the United States. A targeted literature review (TLR) of qualitative and quantitative studies covering HIV and analogous indications (migraine, antipsychotics, and multiple sclerosis) was performed to identify various treatment attributes and their impact on preferences and acceptance of new treatment modalities. A systematic search was performed using Embase. Comprehensive search strings using a combination of Medical Subject Headings (also known as MeSH terms) and free-text words were developed to identify patient and physician preferences of treatment attributes. The final search string utilized for article identification comprised: “disease/indication specific” AND “intervention specific” AND “outcome specific” AND “study design specific.” The search was conducted on April 5, 2023, and was limited to articles published in English between 2013 and 2023. Insights from the TLR were grouped into key attributes and personal factors that hold importance for people living with HIV and for HCPs. Attributes were defined as characteristics of a treatment that individuals consider when evaluating acceptance or preference, and factors were defined as circumstances of an attribute that positively or negatively influence their perspective. Attributes identified in the TLR were used to develop a comprehensive discussion guide for qualitative interviews with both people living with HIV and HCPs. Following attribute selection, pilot interviews informed revisions to discussion guide phrasing and vignette clarity. After sponsor review and consensus, minor amendments were applied, and pilot data were retained in the final analysis as no structural changes to the interview content were warranted.

Interviews with people living with HIV first explored their past and current treatment experiences and then elicited their perspectives on a predefined list of treatment attributes and the factors influencing their preferences, followed by exercises to determine the relative importance they assign to different attributes (see Supplementary File S1, interview questions for people living with HIV). Interviews with HCPs focused on clinically relevant treatment attributes in the context of HIV management and how these attributes influence the benefit–risk trade-offs they encounter when making treatment decisions with their patients (see Supplementary File S2, interview questions for HCPs).

Interviews with both people living with HIV and HCPs focused on five attributes informed by the TLR findings: mode of administration, treatment administration setting, treatment administration frequency, location of injection on the body, and side effects (Table 1). The final 14 interviews with people living with HIV also included the attribute of late-dose leeway, referring to the amount of time a person can delay taking their medication before viral load becomes detectable and the risk of drug resistance increases. Because most modern ART options have high efficacy, efficacy was not included as an attribute. Per the International Society for Pharmacoeconomics and Outcomes Research (ISPOR)-recommended best practice for preference research, this qualitative study identified and validated relevant treatment attributes which will serve as a foundation for a subsequent DCE.

**Table 1:** List of attributes and definitions shown to participants.

Attribute	Definition	Levels
Mode of administration	The way that a medication is delivered to the body	IM injection SC injection IV injection Mixture of IM and IV injection (mixed modality) Mixture of initial IV injection and SC injection (mixed modality) Tablets or pills (oral medication)
Treatment administration setting	The place where a medication is given	HCP-administered injectables Self-administered injectables Administered at home (tablets or pills)
Treatment administration frequency	How often an injection is given	Once in 1 month (self-administered) Once in 2 months (self-administered) Once in 3 months (self-administered) Once in 4 months (HCP-administered) Once in 6 months (HCP-administered) Other
Location of injection on the body	The place on the body where an injection is given	Location Thigh Glutes or buttocks Abdomen Arm
Side effects	Unwanted or unexpected effects that can occur after taking a medication	Redness, swelling, hardness or lumps, itching or rash, or pain at the injection site Discoloration or a change in skin appearance at the injection site Visible marks or nodules from injections Weight gain Diabetes Nausea Vomiting Diarrhea Constipation Other
Late-dose leeway^a^	How much time you can delay taking your medication before your viral load becomes detectable and the risk of drug resistance increases	For injection treatments 2 weeks (for monthly or bimonthly injections) 1 month (for treatments administered once every 3 months, or longer injection treatments) 3 months (for treatments administered once every 4 months, once every 6 months, or longer injection treatments) Other For weekly oral treatments 1–3 days Up to 1 week Up to 2 weeks More than 2 weeks Other

HCP, healthcare provider; IM, intramuscular; IV, intravenous; SC, subcutaneous. ^a^Late-dose leeway was a concept introduced in the final 14 interviews with people living with HIV.

### 2.2 Recruitment and data collection

Potential participants were contacted using a pre-screening form, with recruitment and interviews conducted between August 2023 and January 2024. Individuals were invited to complete an eligibility screener, and those who met eligibility criteria were asked to provide written or electronic consent for their participation. Participants were recruited using a purposive sampling approach and convenience-based identification, with potential participants identified through third-party recruitment vendors. Individuals were screened against predefined inclusion and exclusion criteria, and purposive soft quotas were applied across key characteristics to support inclusion of a broad range of perspectives. For people living with HIV, these characteristics included age, gender, sexual orientation, and prior injection experience. Quotas were monitored through fielding, and recruitment for specific subgroups was closed once target numbers were reached. This approach was intended to capture diversity in perspectives relevant to HIV treatment preferences, rather than to achieve statistical representativeness of the broader population of people living with HIV or HCPs. During the screening process, selection of “male” or “female” was defined as “assigned at birth, on your original birth certificate.” Participants who identified as “transgender/nonbinary” were classified as such. Enrolled HCPs were medically trained clinicians currently treating or managing the care of people living with HIV, with experience treating individuals transitioning to LA injectable ART. The group consisted of five HCPs, with one representative each from the West, Northeast, Midwest, and two representatives from the South regions of the US. Eligibility criteria for people living with HIV included age ≥18 years at study entry and ability to provide confirmation of their HIV diagnosis via clinician-provided confirmation of diagnosis or patient-reported diagnosis of HIV. All participants were required to be US residents and people living with HIV were recruited from the following regions: South (36%), West (32%), Northeast (26%), and Midwest (6%). Participants were also required to be able to read, speak, and understand English or Spanish, and to be willing and able to provide electronic informed consent. Interviews were conducted via telephone by trained and experienced moderators using a semi-structured interview guide. Prior to participant interviews, all moderators completed a training program covering study-specific procedures and standardized interviewing techniques. The training emphasized moderator performance and compliance with study protocols, which was evaluated by senior researchers and/or the qualitative lead. Each interview lasted approximately 60 min. Upon completion of the interviews, each participant was remunerated for their time.

### 2.3 Data analysis

Participant data and interview recordings were managed according to standard data security and privacy procedures. Transcripts were reviewed to redact potentially identifiable information before qualitative coding and analysis. Transcripts in Spanish were reviewed in both the original and translated versions to preserve meaning. Transcripts were then uploaded for qualitative coding and data analysis. Thematic analysis, facilitated by qualitative analysis software (MAXQDA; VERBI GmbH, Berlin, Germany), was employed to analyze transcripts in English or translated to English. To ensure reliability and agreement, we followed a two-phase process: first, 20% of the transcripts were independently double-coded by two coders; second, coded transcripts were compared manually to identify discrepancies, which were then discussed and resolved through collaborative reconciliation sessions involving the coding lead and relevant research team members. To reflect heterogeneous views, saturation was not pursued or applied.

### 2.4 Ethical approval

This study was conducted in accordance with guidelines on good pharmacovigilance practices (European Medicines Agency 2014), preference-based methods from ISPOR Good Practices for Outcomes Research (International Council for Harmonization 1996), applicable regulatory and country-specific requirements (ISPOR 2018), and the Declaration of Helsinki (ISPOR 2017). Central institutional review board approval was obtained from Western Copernicus Group Institutional Review Board (Princeton, NJ) on July 21, 2023, for the following study materials: protocol, discussion guide, confirmation of diagnosis, consent form, screener, stimuli, and outreach and correspondence letters. All participants provided written informed consent before initiation of any study procedures and were compensated for completion of a qualitative interview based on the US fair market values.

## 3 Results

### 3.1 Demographics of interview participants

Interviews were piloted in five people living with HIV and then conducted with 45 additional people for a total of 50 people living with HIV, as well as 5 HCPs in the United States. The people living with HIV identified as male (60%; 30/50), female (38%; 19/50) or transgender/nonbinary (2%; 1/50; Table 2), with a median (interquartile range) age of 50 (39.8–55.8) years. Among the HCPs included, three were physicians, one was a nurse practitioner, and one was a physician assistant (Table 3).

**Table 2:** Demographic characteristics of people living with HIV who participated in the interviews.

Parameter, *n* (%)^a^	People living with HIV (*N* = 50)
Gender	
Male	30 (60)
Female	19 (38)
Transgender or nonbinary	1 (2)
Age, years	
<35	8 (16)
35–44	9 (18)
45–55	20 (40)
>55	13 (26)
Sexual orientation	
Homosexual, gay, men who have sex with men, or lesbian	30 (60)
Heterosexual or straight	17 (34)
Bisexual	3 (6)
Race or Ethnicity	
American Indian or Alaska Native	2 (4)
Asian	1 (2)
Black or African American	17 (34)
Hispanic or Latino	10 (20)
White	17 (34)
Multiple races	3 (6)
US region	
South	18 (36)
West	16 (32)
Northeast	13 (26)
Midwest	3 (6)
Living situation	
Alone	22 (44)
With family members	19 (38)
With friends	1 (2)
With partner or other people	8 (16)
Prior injection treatment experience	
No prior injection treatment experience	31 (62)
Injection treatment experience: other conditions	15 (30)
Injection treatment experience: HIV	4 (8)
Any history of unstable housing	
No history of unstable housing	31 (62)
Some history of unstable housing	19 (38)
History of substance use	
No history of substance use	33 (66)
History of substance use	17 (34)
Comorbidities	*n* = 30
Hypertension	16 (53)
High cholesterol	14 (47)
Anxiety	7 (23)
Depression	6 (20)
Diabetes	5 (17)
Arthritis	4 (13)

^a^Sociodemographic information is reported according to the details provided in the patient-facing document.

**Table 3:** Details of HCPs who participated in the interviews.

Role	Specialty	Years in practice	Setting	People living with HIV treated/year, n^a^	Patients using LA injectable ART
Physician	Infectious disease	31	Urban, HIV specialty clinic	500	1
Physician	Infectious disease	>20	Urban, academic teaching hospital	300	~15
Physician	Infectious disease	40	Suburban, private practice	400	~8
Nurse practitioner	Infectious disease and primary care	>11	Urban, HIV specialty clinic	350	1
Physician assistant	Infectious disease and primary care	>15	Urban, primary care facility	100	Undisclosed^b^

ART, antiretroviral therapy; HCP, healthcare provider; LA, long-acting.

^a^Numbers are approximate.

^b^HCP had experience treating patients with LA injectable ART but did not disclose the number of patients they were treating who are using LA injectable ART.

Of the 50 people living with HIV who were enrolled, 62% (31/50) had no prior injection treatment experience, and 38% (19/50) had prior injection treatment experience for HIV or other conditions. Of the 19 participants with prior injection treatment experience, three had current or prior experience with LA injectable ART. Most people living with HIV (60%; 30/50) reported having at least one comorbidity, including hypertension, high cholesterol, anxiety, depression, diabetes, and arthritis (Table 2). Among those with comorbidities, the majority were managing their conditions with oral treatments, and approximately half also used injectable treatments.

### 3.2 HIV treatment experience

#### 3.2.1 Treatment experiences of people living with HIV

##### 3.2.1.1 Treatment decision-making shaped by HCP guidance and patient experience

Participants across subgroups emphasized trust in their HCP as foundational to treatment decision-making. Most people living with HIV (88%; 44/50) reported that they had switched HIV treatments in the past, primarily guided by HCP advice (73%; 32/44; Table 4). Side effects were the most common reason for past treatment switches (58%; 29/50), with participants describing a range of debilitating experiences, including effects on kidney function, weight gain, vivid abnormal dreams, and gastrointestinal symptoms such as vomiting and diarrhea (Table 4). Bolded text within quotations denotes author-added emphasis for readability and was not present in participant’s original spoken responses.

**Table 4:** HIV treatment experiences of people living with HIV.

Experience	Details	Quotes
Treatment switches	88% (44/50) have switched HIV treatments in the past 73% (32/44) of switches were guided by HCP advice Most common past treatment for HIV: emtricitabine/tenofovir disoproxil fumarate or emtricitabine/tenofovir alafenamide (22/50) Most common current treatment: bictegravir/emtricitabine/tenofovir alafenamide (24/50) 58% (29/50) switched treatments due to side effects Effects on kidney function (16%; 8/50) Vomiting (8%; 4/50) Diarrhea (8%; 4/50) Weight gain (6%; 3/50) Abnormal dreams/nightmares (6%; 3/50)	*“…It was a joint venture in terms of the process that we went through to get to me making the final decision.”* (male, no prior injection treatment) *“…It was a no-brainer. Every day I had to take the pill…I kept telling my doctor, ‘As soon as this injection comes out, I need it’…”* (male, prior injection treatment: LA ART) *“…Diarrhea, daily; every day, diarrhea. I suffered so much. Weak.”* (female, prior injection treatment: other condition)
Oral treatment experience	Approximately half of participants cited convenience as an advantage of oral treatments Daily frequency (30%; 15/50) and remembering to not miss a dose (28%; 14/50) were cited as the most burdensome aspects of oral treatments Convenient dosing schedule (40%; 20/50) and affordability (38%; 19/50) were cited as aspects missing with oral treatments	*“…It’s not really inconvenient for me, and I don’t forget to take it just because I’m in such a pattern…”* (male, no prior injection treatment) *“I think it’s a hassle to take it every day…”* (male, prior injection treatment: other condition) *“I’m tired of having to do that every day, because sometimes I get busy and I forget…”* (male, no prior injection treatment)
Stigma	30% (15/30) reported that stigma affects their decision to keep their HIV status private The majority of participants prefer to take their treatment alone or mostly alone 14% (7/50) do not discuss their treatment with others	*“I don’t like people asking questions, so any time I’m going to have somebody over, I’m going to put all my medication away…”* (male, prior injection treatment: other condition) *“I felt like it was a dirty secret. Yeah, that’s how I felt in the beginning. Now that I am undetectable, and I know how it saves lives and all these medications, I’m more open to speaking about it. I don’t feel uncomfortable talking about it these days.”* (male, no prior injection treatment)
Other treatment challenges	50% (25/50) faced difficulties in accessing medication (getting the medication from the pharmacy, concerns about cost or insurance coverage, securing prescriptions, and medication availability) Several participants reported challenges with adherence 38% (19/50) reported financial/insurance challenges	*“Sometimes the refills run out, and I’m not aware of it. When that happens, sometimes I’ll be out of medication. I have to wait for the doctor’s approval.”* (female, no prior injection treatment) *“Because sometimes I forget to take my pill because I’m too tired, or I get busy around the time. Then I forget to set my alarm clock sometimes, and I just forget.”* (male, no prior injection treatment) *“I’ve had gaps [in] insurances, which made it difficult because I went from having a universal type of…or insurance to having state-run insurance, which made it more difficult.”* (male, prior injection treatment: other condition)
Side effects	Weight gain, gastric issues, and weight loss were the most common side effects experienced with past HIV treatments (n = 3 each)	*“And then you get ready to go somewhere and you feel fine one minute, you take your meds, and that makes you sick to your stomach.”* (male, no prior injection treatment)
Experiences with LA injectable ART	2 individuals were currently using LA injectable ART; another had used LA injectable ART in the past but switched treatment due to lack of insurance coverage Reported advantages: reduced treatment frequency, removal of the daily reminder of HIV status, convenience, privacy, avoiding forgetting to take their pills, not having to worry about dietary restrictions, travel freedom Reported challenges: pain or a mass around injection site, difficulty with insurance coverage, time and cost associated with traveling to their HCP’s office, waiting period after the medication is taken out of storage Aspects to change: ability to self-administer, reduced cost, reduced frequency of injection	*“I don’t have to pick it up, the medicine, anymore. It’s another good thing…No more picking up the pill once…Then every time I’d pick up the pills, every now and then, every couple of months they get new workers there at the [pharmacy], and then those people know my condition now…Those days are over. Then I go to the clinic to get the injection where everybody knows about my condition, so it’s not like it’s a surprise.”* (male, prior injection treatment: LA ART) *“First or second day is very painful. I’m unable to bend down to tie my laces, so I have my roommate help me. Yeah. It’s really painful to bend down.”* (male, prior injection treatment: LA ART) *“It’s such new medication, and insurance agencies just did not want to cover it…”* (male, prior injection treatment: LA ART) “*You have to come there 15 min earlier because they have a storage. They take it out of storage, and it takes 15 min for whatever reason, so it won’t be too cold*.” (male, prior injection treatment: LA ART)

ART, antiretroviral therapy; HCP, healthcare provider; LA, long-acting.

“*All of those adverse reactions that I had. That’s why I switched. Because I had those reactions. I would go from 1 pill that would give me a rash, so I would immediately stop that pill within 3 days and start another one. That pill would give me severe diarrhea and vomiting. I would stop that pill within a week and start another one because **I just couldn’t handle the side effects from those pills***.” (female, prior injection treatment: other condition).

##### 3.2.1.2 Adherence challenges as context, not outcome

Participants reported facing difficulties in accessing medication and other challenges with adherence, which shaped their preferences for simpler regimens. Specifically, participants described a range of structural barriers to adherence, including difficulties coordinating with pharmacies, medication availability, and the burden associated with in-person pick-up. Participants characterized these processes as time-consuming and frustrating, particularly when communication with pharmacies was limited or unpredictable.

*“…you cannot get the pharmacist on the phone when you try to call to make sure that it’s there. That’s very frustrating…Then I have to go in, and then I have to wait. I have to wait for a pharmacist…**I just don’t like the whole process of having to drive to the pharmacy and pick it up.**”* (male, no prior injection treatment).

Personal circumstances, including substance use, were also reported to substantially affect adherence. Participants noted that these situations reduced their ability to follow structured dosing requirements, further compounding the challenges associated with complex regimens.

*“Because when I was on the drugs and the alcohol, I did not stick to my regimen. In those days, it was like, okay, take this pill an hour before you eat. Then you take these 5 an hour after you eat. These ones you take an hour before bedtime. **Somebody who is under the influence has no capacity to understand all those rules and regulations.**”* (female, prior injection treatment: ART).

##### 3.2.1.3 Stigma and privacy

HIV-related stigma emerged as a salient factor shaping how participants managed their treatment in daily life. A substantial number reported taking their HIV treatment alone or actively concealing it from family and friends, reflecting deep-seated concerns about negative judgment and fear of social disclosure.

*“Family wise, [I do not discuss the HIV treatment] not really. The only person who knows is my mom and my sister, so not really…not family wise. **I feel like it’s because of judgment. The stigma that HIV has.** It’s something you really don’t want to go through with your family.”* (male, no prior injection treatment).

Stigma-related concerns also affected treatment access and adherence behaviors. Participants described efforts to avoid being seen obtaining or taking medication, including reluctance to visit pharmacies in familiar environments or concerns about being recognized by others. This illustrates how stigma operates not only at a social level but also in practical, day-to-day decisions about treatment use.

*“I didn’t want to take the pills, pick it up in my neighborhood **in case someone I know in my neighborhood works at that thing.**”* (male, prior injection treatment: LA ART).

##### 3.2.1.4 The emotional value of LA injectables versus the burden of daily oral treatment

A small subset of participants with prior experience using LA injectable ART (n = 3) described a distinct perspective on treatment burden, contrasting the emotional impact of daily oral medication with the perceived benefits of less frequent dosing.

*“Oh, 100%. To me, those couple days each every 2 months, you get a pain there, it lasts for a couple days, it’s okay. I can live with that. That’s nothing. I don’t mind.* ***That’s*** ***a small price to pay.**”* (male, prior injection treatment: LA ART).

For these participants, daily oral treatment was described as a persistent and intrusive reminder of their diagnosis, contributing to ongoing emotional burden. Among those with experience of LA injectable ART, a striking theme emerged around psychological liberation. Cited advantages of LA injectable ART included reduced treatment frequency, removal of the daily reminder of HIV status, convenience, privacy, avoiding forgetting to take their pills, not having to worry about dietary restrictions, and travel freedom.

“*All of a sudden, it’s time to take the pill.* ***It’s like someone tapping you on the back of your shoulder and saying, ‘Uh, you are damaged goods. You’ve got HIV*.’**” (male, prior injection treatment: LA ART).

“***I don’t have to go pick up the medicine anymore.** I don’t have to wait*.” (male, prior injection treatment: LA ART).

“*I could never go to Dubai because I have those medicines. **Now I’m taking the injection, I can go there.** I could beat the system. There’s no way they would know because they don’t take the blood. HIV people were barred from going there.*” (male, prior injection treatment: LA ART).

##### 3.2.1.5 Cost and insurance as spontaneously raised concerns

While LA injectable ART may reduce certain aspects of treatment burden, participants with experience of LA injectable ART spontaneously raised concerns related to broader access and affordability. This included difficulty with insurance coverage, time and cost associated with traveling to their HCP’s office, and a waiting period after the medication is taken out of storage.

Notably, cost and insurance coverage emerged without prompting as priority concerns for a substantial proportion of participants living with HIV, underscoring the significance of these barriers.

Injection-related discomfort (e.g., short-lived pain around the injection site) was generally described as an acceptable trade-off in exchange for reducing the burden associated with daily oral treatment.

“*It’s just a couple of days every 2 months you get that pain. You would think after a while my body would get used to it. Maybe over a year now…Maybe later, like a couple more months, then I’ll only have maybe just 1 day of pain and that’s it. That’s what I think. But if it’s going to continue to be like this, it’s okay. **It’s a trade-off. I don’t care. I’d rather still do this than take the pills every day…I don’t ever want to take the pills again.** I’ve had enough. I’ve [taken] it all those years. I’ve had enough. I’ve had enough.*” (male, prior injection treatment: LA ART).

Despite this overall acceptance, participants identified areas for improvement in LA injectable regimens. These included the ability to self-administer, reduced cost, and reduced frequency of injection.

“*I guess if I could give it in my thigh, I would…that would be great.* ***I could self-administer it like the testosterone*.**” (male, prior injection treatment: LA ART).

*“[Regarding the frequency of injection] **I’d like it to go longer, at least 3 months**, rather than 2*.” (male, prior injection treatment: LA ART).

#### 3.2.2 Findings from HCPs

HCPs expressed overall satisfaction with currently available ART options. Several HCPs commented on the progress made in the number of treatment options available to people living with HIV and mentioned treatment options with less frequent dosing as the primary gap in current HIV treatment.

*“**I think if we can come up with a regimen that was injectable every 6 months**, even if we…if we sort of could sync it with what their expected visits were anyway, I think that would be ideal.”* (Infectious Disease Specialist).

“*[It] would be ideal because if they are already having adherence issues, you know, making sure that they remember it once a month might be more of an issue, and so* ***every 6 months actually might provide some peace of mind that they’re covered for 6 months*.**” (Physician Assistant).

HCPs stated that a minority of people living with HIV in their care were using LA injectable ART, and only 1 HCP reported that they actively approach their patients with this treatment option. The majority of HCPs reported that their patients request the option of LA injectable ART. Patient preference was reported to be the most important driver behind prescribing decisions.

*“Many patients, I mean, it’s a fair mix of both those who are fairly recently diagnosed and who have been living with HIV maybe for many years, so the demographic or the background history on them is a pretty good mix. It kind of just has been their motivation to get them on [LA injectable ART]. T**here are a lot of patients who are really excited about it.**”* (Physician Assistant).

Viral load, insurance coverage, and housing stability were also commonly considered factors in LA injectable prescribing decisions, and 1 HCP mentioned that they were conservative in prescribing novel LA injectable ART until long-term efficacy and safety have been demonstrated. HCPs cited a complex administrative process as the main challenge when prescribing LA injectable ART.

*“It’s just the EMR [electronic medical record], the way they created how to prescribe it.* ***It’s*** ***just very not intuitive.** Pharmacy also gets confused. It’s not easy to do.”* (Infectious Disease Specialist).

### 3.3 Treatment attribute preferences and factors influencing decisions

#### 3.3.1 Ranking of attributes by people living with HIV

After discussing their past and present experiences with HIV treatments, people living with HIV were asked to provide their perspectives on and to rank the importance of six attributes related to HIV treatment when considering a hypothetical treatment switch: mode of administration, treatment administration setting, treatment administration frequency, location of injection on the body, side effects, and late-dose leeway (Table 1). Spontaneously mentioned attributes were also discussed. Of the predefined attributes, side effects were most commonly ranked among the top 3 priorities for people living with HIV, followed closely by treatment administration frequency (Table 5). Mode of administration was ranked third, while treatment administration setting, injection location, and late-dose leeway were assigned progressively lower priority among participants; the latter pertaining to those who discussed this attribute (Figure 1).

**Table 5:** Ranking of attributes by people living with HIV.

Rank^a^	Attribute	Preference	Factors influencing decision	Quotes
1	Side effects of concern	Weight gain was most concerning, followed by diabetes, and adverse gastrointestinal effects; specific injection site reactions commonly mentioned		*“**I wouldn’t want to take a medication that gives me diabetes.** No. That’s trading one issue for the next. No, I do not want that.”* (male, prior injection treatment: other condition) *“**I would be concerned with the redness, swelling, and lumps if they lasted.** If they were just minor and they went away within a few hours or a short amount of time, it’s not a problem.”* (male, prior injection treatment: other condition)
2	Treatment administration frequency		Side effects, cost of medication, travel expenses, and lifestyle	
	HCP-administered	Once every 4 months and/or once every 6 months	Injection coinciding with doctors’ appointments, appointment time/availability, and relationship with HCPs	*“I mean, it says here once every 6 months. That would be the greatest because then that would coincide with all my doctor’s appointments, and **I only have to make one trip for everything.**”* (male, no prior injection treatment) *“I would say once in every 4 months because…**that would be fine with me because I see my doctor once every 4 months** so it would work out just fine.”* (male, prior injection treatment: other condition)
	Self-administered	Once every 3 months or once every 1 month	Storage and disposal, and features of injection device	*“**The automated system of prick,** like a little pin prick, that would be more preferable.”* (male, no prior injection treatment)
3	Mode of administration	Oral tablets/pills (preferred by 28% [14/50] as a stand-alone option, 44% [22/50] preferred options including oral)^b^	Convenient dosing schedule, and side effects	*“**The pills just seem more convenient and less intrusive than an injection.** Again, and I’m going back to my personal lifestyle because I do travel so much, if it was self-administered…it just seems super inconvenient for a person like me.”* (male, no prior injection treatment) *“I do not like [self-administered injections once a month] because I do not want injections. I do not like needles. **I prefer something oral. It’s much easier. I can take it in 2 or 3 s. It’s with water, and that’s it. It’s very easy. There’s no pain.**”* (male, no prior injection treatment)
		Injections (intramuscular preferred by 22% [11/50] as a stand-alone option, 46% [23/50] preferred options including intramuscular injection; subcutaneous preferred by 10% [5/50] as a stand-alone option, 42% [21/50] preferred options including subcutaneous injection)^b^	Side effects (pain), and frequency of medication	*“Just because I feel like the subcutaneous injection is kind of like a TB injection. I never really liked those. Those get itchy, all of those other things. Intramuscular, like you might have a little soreness after the injection, but it typically goes away really quickly and then you do not have to worry too much. **It’s like once it’s done, it’s done.**”* (male, prior injection treatment: LA ART) *“Well, if I were to switch, I think it would be injection under the skin, ideally, or muscle. No preference either way…Well, I think those would be relatively easy to do if I had to self-administer them.* There’s ***no pain involved really. And I feel like with something like that, it would be a lot less frequently than taking a pill once a day.**”* (male, prior injection treatment: other condition) *“I guess it would be the dosage. How many times? Is it less? Is it more? **I guess if it was something that I do once a week or once a month, I would definitely switch**.”* (female, no prior injection treatment)
4	Treatment administration setting		Cost, and availability of patient support services	*“[I’ll] Do it myself…Because I don’t have to deal with going out and going to a clinic, a medical center, or whatever. No, **anything I can do at home, anything to make life easier,*** that’s me.” (female, prior injection treatment: other condition)
		Self-administered injection setting (preferred by 64% [32/50])	Storage and disposal, features of injection device, and convenience	*“**It would have to be easy to use** because, if this is something that you want people to pick up on and start using and stuff…I would like it to be really easy, something that I really do not have to think about. Pictures included. Along with written instructions and somebody to teach me how to do it.*” (female, prior injection treatment: ART)
		HCP-administered injection setting (preferred by 58% [29/50])	Ease of scheduling and duration of appointments, discomfort or lack of experience with self-injection, and trust in HCPs	*“**Follow-up to see how you’re doing after the service.** See if it worked, if it did not work, are there any side effects, do they need anything else. If there’s redness, they need some alcohol rubs or something, but to see how they are doing after the fact.”* (female, no prior injection treatment)
5	Location of injection on body		Convenience of body location, and side effects (mainly pain)	*“I feel like being sore in my arm, I can take.* Versus *being sore in my abdomen sounds weird. Regardless, **those injections make you sore…So you want to be sore in an area that is a little bit convenient.**”* (transgender/nonbinary, no prior injection treatment)
	HCP-administered	Arm (preferred by 80% [40/50]), followed by glutes or buttocks (preferred by 62% [31/50])	Time (e.g., time between injections, waiting time)	*“If there’s some downtime in between the injections, chances are, if you have to wait for 15 min, chances are, at the doctor’s, you are going to be waiting over 15 min…so that makes your…**it makes staying at your doctor a lot longer.**”* (male, prior injection treatment: other condition)
	Self-administered	Abdomen (82% [41/50] preferred single abdominal injection; the majority of participants preferred 2 injections)	Device ease of use, fear of needles, and privacy/discretion of the device	*“With that type of injectable it seems like it would be like a diabetic pen thing injectable, so that’s pretty private to me. I can easily go to the bathroom and do that or go to another room and give myself an injection. **It would not be to where I would worry about other people seeing me with it.**”* (female, no prior injection treatment)
6	Leeway			
	Minimum acceptable leeway	Ranged between 1–3 days (preferred by 6/14 participants) and 1 month (preferred by 2/14 participants); 5/14 preferred 2 weeks; 1/14 did not provide their perspective	Efficacy concerns, viral suppression, and treatment shortages	*“I just do not think I should take a leeway off. I don’t want to take off. If I’m actively doing this and I’m consistent, **I want to stay consistent.**”* (male, no prior injection treatment)
	2-week leeway for monthly or bimonthly injections	Acceptable to 8/14 participants; not acceptable to 1/14; 5/14 did not provide their perspective	Personal experiences with adherence, and resistance	*“**I probably would not consider an injectable where I had to go to a doctor’s office, a non–self-administered injectable, if it did not have that 2-week…wiggle room.** Because I do travel or I’m maybe not feeling well.”* (male, prior injection treatment: other condition)
	1-month leeway for treatments administered once every 3 months	Acceptable to 7/14 participants; not acceptable to 3/14; 4/14 did not provide their perspective	Lifestyle choices, travel, and mode of administration	
	3-month leeway for treatments administered once every 4 or 6 months	Acceptable to 6/14 participants; not acceptable to 4/14; 1/14 uncertain; 3/14 did not provide their perspective	Consistency, adherence, and efficacy concerns	*“That, I’d be kind of leery on, for every 4…to miss something by 3 months when it’s due every 4 months. **I would be kind of leery about taking away the effectiveness of it or drug resistance increases.** I’d be leery of that.”* (male, prior injection treatment: other condition)

ART, antiretroviral therapy; HCP, healthcare provider; LA, long-acting; TB, tuberculosis.

^a^Attributes were ranked in response to the following prompt: “Would you please rank these attributes [presented based on prior discussion] when deciding whether to switch or not to switch to a future HIV treatment from most important to least important, where 1 indicates the most important and [5, 6, or total number of factors] indicates the least important?”.

^b^Preferences include stand-alone options (oral tablets/pills [28%], intramuscular injection [22%], subcutaneous injection [10%], and intravenous injection [4%]) and combined options (either intramuscular or subcutaneous injection [16%]; either subcutaneous injection or oral tablets/pills [10%]; either intramuscular, subcutaneous, or intravenous injection [4%]; either intramuscular injection or oral tablets/pills [2%]; either intravenous injection or oral tablets/pills [2%]; and either intramuscular or subcutaneous injection or oral tablets/pills [2%]).

**Figure 1:**
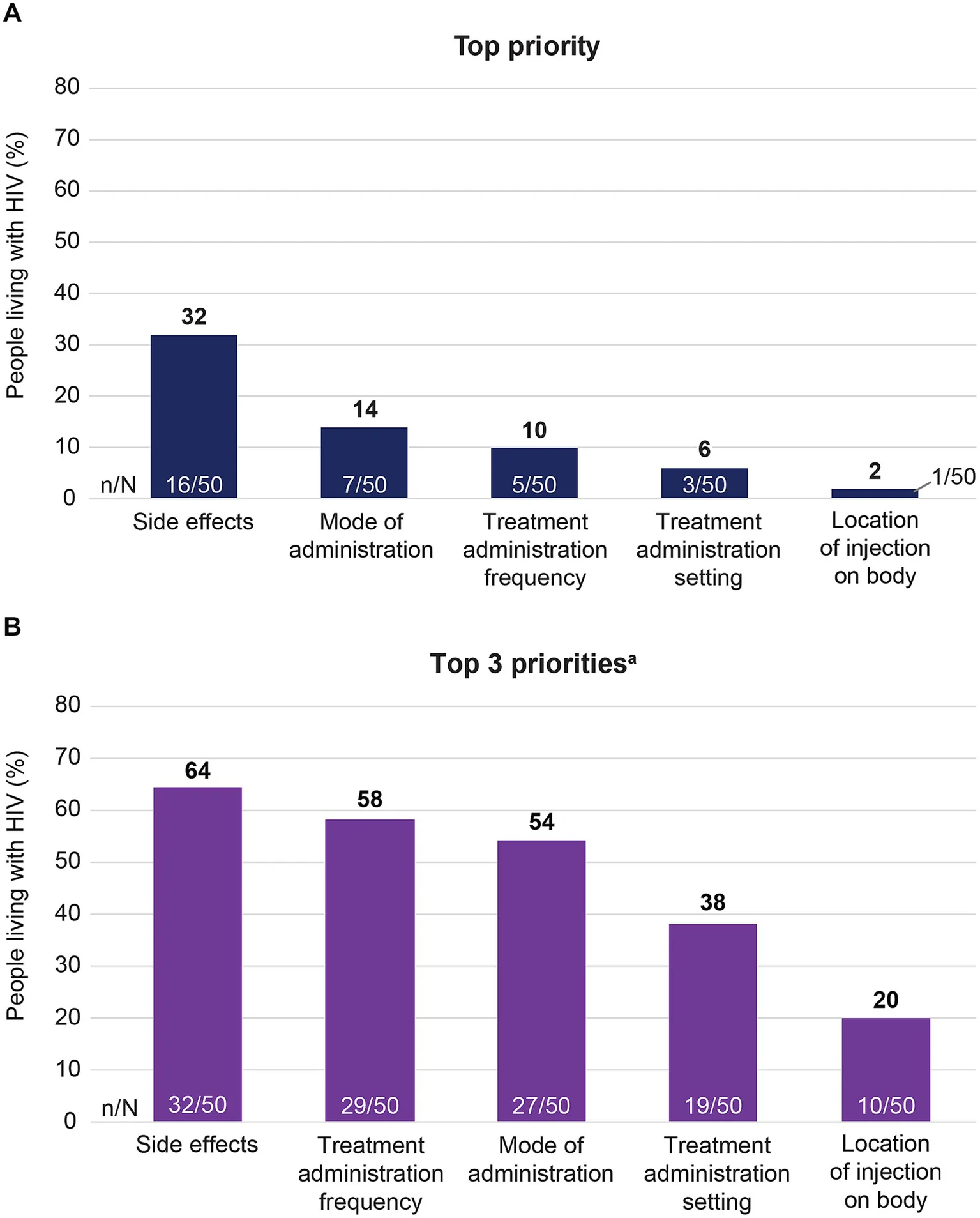
**(A)** top priority and **(B)** top 3 priorities ranked by participants when considering treatment switch. Participants ranked each predefined HIV treatment attribute in response to the following prompt: “Would you please rank these attributes [presented based on prior discussion] when deciding whether to switch or not to switch to a future HIV treatment from most important to least important, where 1 indicates the most important and [5, 6, or total number of factors] indicates the least important?”. ^a^Late-dose leeway ranked among the top 3 priorities of 1/14 (7%) participants.

Beyond the predefined attributes, cost emerged as the most prominent spontaneously mentioned concern, with a notable number of participants ranking it among their highest priorities (26%; 13/50). Number of injections, insurance coverage, paperwork burden, and privacy of medication access were also raised by participants, reflecting the practical and structural dimensions of treatment access that preoccupied many.

When all injectable forms were considered together, most participants expressed a preference for injectable over oral administration, although no single injectable modality individually surpassed oral tablets/pills in preference. Intramuscular and subcutaneous injections attracted broadly positive responses, with many participants perceiving the latter as less painful and more suitable for self-administration. Preferences for oral administration were primarily grounded in familiarity, simplicity, and the absence of needle-related anxiety.

*“Well, my preference would be oral medication. But I mean, that also takes into account of if **I’m able to do it by the other means less frequently, a lot less frequently, then I am open to doing.**”* (male, prior injection treatment: other condition).

*“Let’s say I talked to my doctor and there’s this new HIV med that’s a shot once every 2 months. I would be quicker to embrace it if [it] was subcutaneous than intramuscular, but once every 2 months I can deal with an intramuscular shot…I mean, intramuscular shots are not fun to give yourself, but **I would do it to make my overall HIV treatment less burdensome.**”* (male, prior injection treatment: other condition).

Nearly all participants expressed a clear preference for HCP-administered injections at 4- or 6-month intervals, with this preference consistently anchored in practical considerations: alignment with existing medical appointment schedules, appointment availability, and the quality of ongoing HCP relationships. For self-administered injections, preferences were more distributed, though quarterly administration attracted the strongest interest. People living with HIV who had a history of unstable housing showed a preference for an HCP-administered injection frequency of once every 6 months versus once every 4 months. Participants who prioritized convenience and reduced clinic visits also tended to prefer injections every 6 months. However, other participants preferred every 4 months, citing regular health monitoring (e.g., viral load and undetectability checks) as a key reason for this choice. Some participants also expressed concern about the potential impact of higher medication doses required for 6-month intervals, leading some to lean toward the shorter 4-month frequency. Side effects, medication cost, travel expenses, and lifestyle were commonly considered important factors regardless of who administered the treatment.

*“I would go as low as monthly. But of course, once every 3 months would be the best. **Not having to worry about having to do the pills daily, I would do a self-administered injectable monthly.**”* (male, prior injection treatment: other condition).

Regarding frequency of oral HIV treatment, the large majority of people living with HIV preferred weekly over daily administration. Participants expressed a strong desire for less burdensome treatment regimens and associated fewer side effects with less frequent administration.

*“**It might be easier to manage once a week.** I mean, I do it anyway, but I think it would get easier to manage. I don’t know. Maybe that would also reduce it somewhat. I don’t have them but it’s reducing the amount of… I don’t know if it reduces the amount of toxicity to the body because you’re having to put it in less times.”* (female, no prior injection treatment).

Some participants also expressed that less frequent administration may improve adherence and quality of life.

*“Weekly, absolutely…Because it’s once a week. I mean it’s less pills. It’s less hassle. **It’s less forgetting. It’s less everything.** The less the pills, the better for me.”* (female, prior injection treatment: other condition).

*“I wouldn’t have as many pills to finagle with and manage. **Instead of 30 pills in a month, it would be 4. It would…be kind of out of sight, out of mind**…I’d feel like once a week is a very easy thing to handle and put in my pillbox…Cutting down on it, the amounts, would be wonderful.”* (male, prior injection treatment: other condition).

Preferences for HCP- versus self-administered injections were notably heterogeneous, with the sample broadly divided between both options and a subset expressing openness to either depending on context. Rather than a single dominant preference, the data revealed that administration setting choices were highly contingent on individual circumstances, prior injection experience, housing stability, trust in HCP, and lifestyle. Cost and availability of patient support services were common important factors of consideration regardless of administration setting.

“*I would want to consider [patient support services] to a certain extent. Currently I don’t have to even consider that because it is so easy to take my current medication that I literally just take the pill myself. I don’t have to worry about asking any other questions or having any issues with that. **If it were an injector, I would want some type of convenient chat function, live chat function to where I’m not speaking with an auto bot, but I’m speaking with an actual healthcare professional if I have any issues**…*” (male, no prior injection treatment).

The treatment administration preferences of people living with HIV were driven by trust in HCPs and lifestyle compatibility. Preferences for HCP-administered injections were contingent on ease of scheduling and duration of appointments, lack of comfort or experience with self-injection, and trust in HCPs.

*“Because if it’s hard to schedule and you can’t really get one, **you are missing treatment**.”* (female, no prior injection treatment).

*“Not only the fear of needles, but also because of that fear of needles, **am I going to administer it correctly?** I do not want to mess up my veins or body if I’m not doing it properly by having that fear…”* (male, no prior injection treatment).

“*Instead of me doing it on my own…because I’m not trained as a healthcare professional. I’m just me, as a patient, just doing it on my own rather than **a healthcare professional properly doing it to me in their presence, in their expertise.** I think that’s very important*.” (male, no prior injection treatment).

Preferences for self-administered injections were contingent on storage and disposal, features of the injection device, and convenience.

“*The ease of use and convenience [of the device] would be slightly important. Ozempic, I found to be pretty easy to use. I just watched a short video on it, and I was able to understand. I think I’m capable of learning anything.* ***Overall, ease of use would be important but I don’t think it would be that difficult*.**” (male, prior injection treatment: other condition).

Side effects were a major factor shaping the treatment preferences of people living with HIV. Participants had experienced side effects with current and past oral HIV treatments and wanted to avoid side effects with future HIV treatments.

*“Because you have to live your daily life with the side effects. If you have severe side effects, you cannot function as a normal human being. And **you want to be able to take your medicine and live your life to the fullest when you are in this situation**. I know what it’s like to have side effects and not be able to live like a normal person. So that’s most important to me.”* (female, prior injection treatment: other condition).

*“I couldn’t hold any food inside of my system. Literally, **I couldn’t keep anything inside of me. I had horrible fatigue, dizzy spells a lot.**”* (male, prior injection treatment: LA ART).

*“Well, **I had so many side effects from so many medications** that…yeah, that’s important.”* (female, prior injection treatment: other condition).

Among probed side effects, weight gain, diabetes, and adverse gastrointestinal effects were of comparable and widespread concern across the sample. Visible skin changes at the injection site, such as red marks and nodules, were also raised by many participants but were generally regarded as less concerning than the systemic effects, particularly when they were expected to resolve quickly.

*“**Nausea, vomiting, [diarrhea], constipation, those are not pleasant.** I had those with [tablets]. I would not want that again.”* (male, no prior injection treatment).

*“Well, if it’s going to be on something…a visible part of my body with side effects, **I wouldn’t want to have a rash or something that stands out or some kind of lump or something like that, where I’m going to be questioned as to what it is** or something that’s going to be annoying that I’m going to have to scratch. That would be important.”* (male, prior injection treatment: other condition).

For HCP-administered injections, most participants preferred the arm as the injection site, while the abdominal region was broadly preferred for self-administered injections. No differences in preferred location of injection on the body were observed across subgroups of participants with prior injectable experience, history of unstable housing, or prior substance use. Convenience, familiarity, and minimizing visible evidence of injection were the primary factors shaping these preferences. When two sequential injections were required, participants generally favored a brief waiting period between them, expressing concern that longer delays would unduly extend their clinic appointments.

*“15 min would be ideal. **20 min, that would just be pushing it for me because that would just be too long out of my day once again.**”* (male, no prior injection treatment).

People living with HIV were asked for their preferences regarding late-dose leeway for injection treatments and were offered options of 2 weeks for monthly or bimonthly injections, 1 month for injections administered once every 3 months or longer, 3 months for injections administered once every 4 months or longer, or any other options that were spontaneously mentioned. For late-dose leeway with weekly oral treatments, participants were offered options of 1 to 3 days, up to 1 week, up to 2 weeks, more than 2 weeks, or any other options that were spontaneously mentioned. People living with HIV had different perspectives on late-dose leeway, with the acceptable minimum leeway ranging from 1 day for weekly oral treatments to 1 month for injection treatments. Participants reported efficacy concerns, viral suppression, and treatment shortages as the main factors of consideration in their choices regarding minimum acceptable leeway.

#### 3.3.2 Ranking of attributes by HCPs

HCPs were also asked to provide their perspectives on and to rank the importance of a list of five attributes related to HIV treatment when considering a treatment switch: mode of administration, treatment administration setting, treatment administration frequency, location of injection on the body, and side effects. Among the predefined attributes, HCPs ranked side effects and treatment administration frequency as the most high-priority attributes when considering a treatment switch (Table 6). Mode of administration was consistently ranked third by all HCP participants, and treatment administration setting and location of injection on the body were generally ranked as lower priority. Other attributes and factors spontaneously mentioned by HCPs included patient preference, adherence history, comorbidities, and housing stability.

**Table 6:** Ranking of attributes by HCPs.

Rank	Attribute	Preference	Factors influencing decision	Quotes
1	Side effects of concern	Weight gain, injection site reactions, and nodule marks were most concerning		*“**Weight gain is definitely an issue for our patients.** I would say that’s probably the most common reason patients ask to be switched, so that’s probably number 1 on my list of those there.”* (Physician Assistant)
2	Treatment administration frequency			“*I think patients who would be willing to have injections, I think they would all be very open minded to something that would be every 6 months. It’s like I said. Whenever we have these conversations—and these are often patient-initiated conversations—**the biggest point that they’ve all got is the frequency. When they talk about it, they say, ‘Why can’t they come up with something that’s every 6 months when I come in anyway?’** So, I think that would be embraced by patients. Patients who would be willing to take injectables.*” (Infectious Disease Specialist)
	HCP-administered	Once every 6 months	Adherence, patient preference, and level of virologic suppression	*“**The less frequent, the better.** So, we started off where [LA injectable ART] was available every month. Nobody was interested. When it became every 2 months, all of a sudden, a lot of people became interested…these are often patient-initiated conversations—the biggest point that they have all got is the frequency.”* (Infectious Disease Specialist)
	Self-administered	Varied preferences	Adherence, patient preference, injection administration, and education	*“So here you’d be having frequency of injectables at home compared with oral daily therapy, which is typically 1 reasonably sized pill a day…I don’t know when it would be where patients would go for it…maybe at once a week, they might be interested. Maybe more at once a month, more at every 2 months. **I think the less frequent it is, the more you’d have who would embrace it.** But there might be some even if it was a once weekly injection might embrace it, if you compare it with a daily oral.”* (Infectious Disease Specialist)
3	Mode of administration	Oral	Patient preference, and adherence	*“The question is whether they’ll remember a weekly oral versus a daily oral, and will that affect compliance?...if you were on other medications for whatever, what’s another medication? What’s another pill to you? You just threw down 5 pills, what’s 6 pills?…but again, **it all boils down to what the patient wants and what he or she can do effectively.**”* (Infectious Disease Specialist)
		Subcutaneous	Ease of administration, and minimal pain	*“**I feel like patients feel a little bit more comfortable with a subcutaneous injection.** It’s typically a little bit more comfortable for them as far as tolerability and pain.”* (Physician Assistant)
		Intramuscular	Pain, and patient weight	*“I know in the early studies, there was problems with patients with very high BMIs, where **there was concern about whether or not the medication was being adequately distributed.** And then you’d have to worry about patients who are very thin, who do not have muscle mass.”* (Infectious Disease Specialist)
		Intravenous	Complicated, and unsuitable for people with history of substance use or poor venous access	*“Intravenous is not painful, but the insertion of the IV, which is going to take more time and be more complicated with the nurses. **If the patient does not have good veins, then that could take…it may not even be an option** or it could take a long…tie up a nurse for a long time.”* (Infectious Disease Specialist)
		Mixed modality	Complicated, offers little incentive to patients, and adherence issues	*“Because the oral and the injectable will be very confusing. The patient will ask, ‘Why do I have to take both?’ If the oral medication…if the patient only takes one a week and then the injection one a month, something there…But **it would be very confusing for the patient.**”* (Nurse Practitioner)
4	Treatment administration setting	Self-administered injection setting	Personal factors: patient preference, patient history of substance use, and patient housing stability Treatment attributes: adherence, mode of administration, ease of administration, clinic resourcing, storage, and administration training	*“For my patients who are really adherent and have all of the kind of social supports and stabilities, I think that just creates, again, **that ease of use where they’re not even having to come into the clinic to get their injectable,** again, to have that reminder or extra appointment to care for their HIV. I think that would be a great option for some of my patients.”* (Physician Assistant)
		HCP-administered injection setting		*“One of the things about bringing people back for follow-up is **they may be doing fine on a single tablet and they come in every 6 months, but that’s not enough to screen them for STDs** because you string it out that long, you are going to miss STDs, and we do miss STDs because the HIV side is easy to treat, but the STD screening is more problematic.”* (Infectious Disease Specialist)
5	Location of injection on body		Mode of administration, injection pain, injection site reactions, familiarity with injection location, convenience of body location, and ease of administration	*“I do think it would play a part with self-administer just because if I have a patient who lives alone, especially, something like a glute or buttock is more difficult to inject on their own. I think that would play a part certainly in that aspect of it…**I still think patients would overall prefer a subcutaneous abdominal injection overall.**”* (Physician Assistant)
	HCP-administered	Glutes and arm		*“**I think it would be an area that hurts less, I guess.** As far as looking at the easy areas, probably buttocks is going to be the one that hurts the least.”* (Infectious Disease Specialist)
	Self-administered	Abdomen		*“**I think most people would like to get something that they can administer, and that would probably be the abdomen.** I think that would be the easiest for somebody to inject, **just like insulin or other drugs where you can do it yourself.**”* (Infectious Disease Specialist)

ART, antiretroviral therapy; BMI, body mass index; HCP, healthcare provider; IV, intravenous; LA, long-acting; STD, sexually transmitted disease.

When considering mode of administration, patient preference was the most frequently mentioned factor by HCPs. Oral treatment was regarded as the simplest mode of administration, although HCPs had mixed preferences for daily versus weekly administration of pills. Some HCPs expressed concerns regarding adherence to weekly pills.

“*We’re used to taking daily medications by mouth, so that’s why we would probably prefer that, but would it be simpler for some to take it once a week and they were compliant with that, maybe, but again,* ***it all boils down to what the patient wants and what he or she can do effectively*.**” (Infectious Disease Specialist).

Among injectable modes of administration, HCPs overall regarded subcutaneous injection to be the most suitable for people living with HIV, citing ease of administration and minimal pain.

*“**I just think ease of use.** I feel like patients feel a little bit more comfortable with a subcutaneous injection. It’s typically a little bit more comfortable for them as far as tolerability and pain.”* (Physician Assistant).

Regarding frequency of HCP-administered injections, most HCPs preferred the least frequent injection option (once every 6 months) and concluded that scheduling injections during regular check-up appointments would be preferable. Preferences of HCPs regarding frequency of self-administered injections varied.

*“Once my patients have been stabilized, I’m typically seeing them about every 6 months, so **I think a 6-month option is great because then they would come in for their appointment, see me, and they’d get their injection the same day.**”* (Physician Assistant).

“*Basically, the longer the interval, the better it’s going to be and the more likely, I think, the patient will opt for it. So, when you look at this list here, it would just go in the reverse order in terms of preference. 6 better than 4 better than 3 better than 2. I don’t think anyone would opt for 1 month if you could do 2 months, with [LA injectable ART], I mean. So, it’d just go in reverse.* ***The longer interval, the better it is*.**” (Infectious Disease Specialist).

A self-administered injection setting was preferred by most HCPs for their patients. However, HCPs expressed the caveat that self-administration would only be suitable for individuals who demonstrate a consistent history of treatment adherence. One HCP reported that they preferred an HCP-administered injection setting, citing concerns with the increased likelihood of missing other infections when people living with HIV reduce their visits to the clinic.

Weight gain, injection site reactions, and lumps or swelling under the skin at the injection site were the most common side effects of concern among HCPs.

*“Side effects are always an issue, right? Like I said, **when you make a treatment decision, efficacy is always the first and side effects would probably be second.**”* (Infectious Disease Specialist).

*“**Some of the oral medications have been implicated with weight gain.** It’s a big concern…”* (Infectious Disease Specialist).

## 4 Discussion

This qualitative interview study identified treatment attribute preferences and factors of influence among people living with HIV and among HCPs in the United States. Both groups considered side effects, treatment administration frequency, and mode of administration as the most important attributes when contemplating a treatment switch. Mode of administration options, including injections, attracted notable interest among people living with HIV, with most participants expressing preference for injectable options when considering all forms together (including intramuscular, subcutaneous, and intravenous injections, and combinations of these options). Notable interest in injectable modes of treatment administration among people living with HIV was also reported in another recent study, although this interest in LA injectable ART decreased if it had to be paired with oral ART (16). People living with HIV tended to prefer longer intervals (4–6 months) for HCP-administered injections and shorter intervals (3 months) for self-administered injections. Preferences for LA or less frequent treatment should not be interpreted as universal, as some participants, particularly those accustomed to daily medication routines or taking oral treatments for comorbidities, may find daily oral ART manageable. Interestingly, a bimonthly interval was cited as a barrier to switching in a Swiss cohort study, where disruption of an established daily routine outweighed the appeal of reduced dosing frequency for some participants (17), underscoring that interval preferences are highly individual rather than patients uniformly favoring longer intervals. However, the more prevalent preference for longer treatment intervals is in line with findings from a recent study in which 88% of people living with HIV stated that they would be interested in switching to an injectable ART regimen administered every 6 months (16). HCPs preferred every-6-month dosing intervals for injections to align with the appointment schedules of their patients with stable health, although it is possible that in a larger sample, a higher proportion of HCPs see their patients every 4 months rather than every 6 months. Weight gain was among the side effects of most concern both to people living with HIV and to HCPs, and injection site reactions were also frequently mentioned by both groups. These priorities did not emerge in isolation; they were shaped by deeply personal experiences of living with HIV, including the burden of managing daily regimes, concerns about stigma and privacy, and the relational trust that individuals place in their HCPs. Understanding these contextual drivers is essential in interpreting what the ranked preferences actually reflect.

Consistent with other qualitative and quantitative preference studies in the United States (12, 18–20), side effects were among the most important treatment attributes considered by people living with HIV, particularly weight gain, diabetes, and gastrointestinal effects. Treatment frequency was also deemed an important attribute among people living with HIV in some previous studies (18, 21), although it only significantly impacted treatment choices of HCPs but not people living with HIV in another study (12). Preferences for treatment attributes among people living with HIV were often similar to those of individuals with other health conditions that have both oral and LA injectable treatment options (22, 23). In the present study, treatment administration frequency was ranked as the second most important predefined HIV treatment attribute, but it was ranked as least important in a patient preference study on migraine treatment attributes, in which mode of administration was ranked first, followed by durability of effectiveness (24).

Regulatory agencies such as the US Food and Drug Administration seek to understand the treatment-related benefit and risk trade-offs that are acceptable to patients, and patients’ direct experiences with available treatments play an important role in regulatory decision-making (14). Preference findings in the present study can support regulatory understanding of HIV treatment options and contribute to maximum acceptable risk, patient care, and post-authorization assessments. For HTA bodies and payers, preference evidence can also support identification and weighing of endpoints, as well as indicate which subgroups within a larger population might particularly value a treatment. Patient preferences regarding treatment options may have important cost-effectiveness implications, as these preferences may affect adherence and clinical outcomes, and it has been suggested that payers should include patient preferences in their cost-effectiveness models (4). Importantly, this study identified cost and insurance coverage as spontaneous responses and primary concerns, suggesting these variables may be insufficiently captured in studies relying exclusively on predefined attributes. The salience of cost and insurance reflects a broader pattern observed throughout the interviews: participants weighed treatment options against the realities of their daily lives, not only against clinical attributes in the abstract. Further, preference evidence can support treatment uptake among HCPs and promote patient-centricity and shared decision-making in clinical practice. Our findings provide real-world patient voice data that directly inform patient-centered care and shared decision-making. This study also revealed the contextual nature of preferences, where choices were contingent on factors such as housing stability, stigma, HCP trust, and prior treatment experience. These contextual dimensions underscore the value of qualitative methods in capturing what structured approaches may not readily surface. While the present findings are specific to the US context, a comparison with a non-US cohort offers a useful reference point. The Swiss cohort study cited above similarly reported high satisfaction with oral ART and identified individualized, rather than uniform, preferences for treatment attributes among people living with HIV, suggesting some consistency in preference patterns across health systems (17). However, awareness of LA injectable options differed notably by gender and age in the Swiss cohort, a dimension not directly assessed in the current qualitative study but worth considering in future work, given the contextual and relational factors (e.g., HCP trust) identified herein. These findings will inform the development of a subsequent DCE to quantify preferences and estimate trade-offs across treatment attributes.

### 4.1 Limitations

There are a few limitations to consider. First, the convenience sampling method used may limit generalizability and is likely to have introduced selection bias. Generalizability may be further limited by the small sample sizes within subgroups. Relatedly, illustrative quotations may not fully capture the breadth of participant perspectives, particularly among those who considered daily oral ART acceptable. The apparent underrepresentation of the illustrative quotes may reflect small subgroup sizes, convenience sampling or selection bias, quote-selection constraints, or a combination of these factors. In addition, HCPs in our study were restricted to those practicing in urban and suburban centers, which may not fully capture structural barriers and resource constraints experienced by rural practitioners. The study also relied on preferences regarding a hypothetical treatment switch, which may overestimate willingness to adopt new treatment modalities without fully considering cost, access, or other constraints. Efficacy or effectiveness was not included as a predefined ranking attribute because currently marketed ART options were considered to have broadly similar effectiveness; therefore, the study could not directly assess how participants valued this characteristic relative to other treatment attributes, despite its established importance in ART preferences and HCP decision-making. Efficacy-related concerns emerged indirectly through discussions of viral suppression, drug resistance, adherence, and late-dose leeway, but the omission of efficacy may have influenced the relative importance assigned to other attributes and should be considered when interpreting the findings. The subsequent DCE will use forced trade-offs to reduce hypothetical bias and provide more precise preference estimates. Lastly, participants’ subjective interpretation of treatment attributes, recall bias, and social desirability bias could have influenced their responses, potentially leading to inaccuracies. However, the inclusion of pilot interview data strengthens the sample size and reflects the iterative, collaborative development process undertaken to ensure instrument validity prior to full data collection.

## 5 Conclusion

This qualitative interview study provides valuable insights into the preferences of people living with HIV and of HCPs regarding HIV treatment attributes as well as the factors influencing these preferences. This study is, to our knowledge, one of the few to explore the full range of HIV treatment attributes (beyond injection versus oral comparisons), capturing both predefined and spontaneously mentioned attributes, with clinically actionable subgroup analyses that are absent from existing DCE literature. New treatment options that take the preferences for fewer side effects and reduced treatment administration frequency into account may improve quality of life and treatment adherence of people living with HIV. A planned quantitative DCE will assess generalizability of these findings in a larger population with broader geographical reach and provide additional evidence of preferences for regulatory and HTA bodies.

## Data Availability

The original contributions presented in the study are included in the article/Supplementary material, further inquiries can be directed to the corresponding author.
