# Supplementary material for: Preferences of people living with HIV for treatment regimen attributes: a qualitative interview study
Source: Front Public Health. 2026 Sep 14;14:1907747. doi: 10.3389/fpubh.2026.1907747 (PMC13617073; doi:10.3389/fpubh.2026.1907747)
Supplement: Supplementary file 1 [file Data_Sheet_1.pdf]

Supplementary File S1. Patient Preferences for Regimen Attributes for Treatment of HIV, November 2023.

Patient Preferences For Regimen Attributes For Treatment Of HIV

-- PWH Discussion Guide –

November 15th, 2023

DOCUMENT'S SPECIFICATIONS

**TITLE**	**Patient Preferences For Regimen Attributes For Treatment Of HIV – PWH Discussion Guide**
**VERSION**	**V.05**
**DATE**	**November 15th, 2023**
**CONDUCTED BY**	**IQVIA**

DOCUMENT'S PURPOSE

This discussion guide is designed for conducting semi-structured interviews with people living with HIV (PWH). The objective of the interview is to collect qualitative data regarding PWH preferences for long-acting injectable and oral antiretroviral therapy (ART) when switching from daily oral therapy. **The interview aims to explore the various factors that influence PWH preferences for different long-acting injectable options, as well as the significance of regimen attributes for PWH.** Additionally, it will examine the trade-offs faced by PWH when considering different treatment options and assess the level of patient-reported satisfaction with modern ART regimens. The interview also seeks to identify any unmet needs that may exist in the context of HIV treatment.

INTERVIEW STRUCTURE AND TIMING

Interview with PWH participants		
**Guide section**	**Objective**	**Timing (min)**
1. Introduction and disclosures	Explain the purpose of the interview, obtain informed consent, and disclose relevant study information.	2
2. Background information	Gather socio-demographic information about the participant.	5
3. Concept elicitation of experiences with disease and treatments	Understand the participant's experiences with HIV, including treatments and impact on daily life.	20
4. Screenshare set-up	Ensure participant is connected to the platform used for the interview so that they can see any information shown by screensharing.	0.5
5. Description of treatment attributes to capture preferences	Capture the participant's thoughts, and preferences regarding each treatment attribute.	20
6. Ranking of attributes	Engage the participant in ranking the importance of treatment attributes based on their preferences and priorities.	5
7. Treatment scenarios or vignettes	Facilitate a deeper exploration of factors that influence PWH perspectives on ART-related risks and benefits.	5
8. Wrap-up	Provide the participant with an opportunity to share any additional insights or concerns towards HIV and long-acting treatments.	2.5
	**Total**	**60**

1 INTRODUCTION AND DISCLOSURES (2 MINUTES)

[Note to reader – the text here is intended to be a "guide" and not to be read verbatim. An experienced moderator will plan to cover these topics but may use alternative language depending on the natural direction of the conversation.]

Hello. This is **[Moderator Name]** from IQVIA for our scheduled interview. This interview will take approximately 60 minutes. Is this still a good time to talk?

[If no] – Is there another time that works for you? **[Reschedule as appropriate]**

[If yes – proceed below]

As a reminder, the purpose of this 60-minute interview study is to speak with PWH, as yourself, in order to better understand your preferences when it comes to HIV treatments. The interview will be divided into two sections:

1. In the first section, we will be discussing your experience with HIV and how this impact you and your everyday life. Majorly, we will be asking about your thoughts on certain aspects of HIV treatment and to understand deeply how much they matter to you and how they impact your decision making. In addition, we will explore your opinions towards key treatment characteristics, how important these characteristics are to you and the trade-offs between these characteristics.
2. In the second section, we will ask you to rank and rate treatment characteristics depending on your preferences. Next, we present you with some hypothetical scenarios related to HIV treatments and will ask your feedback related to how well you understand each scenario, what characteristics are missing and how it can be improved.

If you have any symptoms, health issues or other concerns, please be sure to discuss these with your doctor or nurse. The answers you provide to the questions in this interview are for research purposes only and are not being shared with the members of your healthcare team. You are free to share anything we discuss today with your doctor if you wish.

We understand that some topics may be sensitive, so please feel free to let us know if you're uncomfortable answering any questions. Your privacy is important to us, and we'll make sure to respect it throughout the interview.

The plan is to audio-record this conversation today so that I am able to focus on our conversation and create a transcript of the interview after. Confidentiality is very important to us, so once we start recording, please do not use your name or the names of family, friends, or doctor / medical personnel. If you mention a name accidentally during the interview, we will remove it from the transcript to preserve your anonymity. Additionally, if we are connecting with you through a conferencing platform on a computer or mobile device, kindly ensure that your webcam remains disabled throughout the duration of the interview.

Any reports that result from these interviews will include data where all personally identifying information has been removed and / or reported at a combined level, which means that it will not be possible for you to be individually identified.

If you report an adverse event (for example, a side effect of a medication) related to any of the study Sponsor's products, IQVIA is obligated to report this. If that occurs, are you willing to have your name and contact information sent to the Sponsor <and the site principal investigator> so that it can be passed on to the Sponsor's drug safety department for the express and sole purpose of follow-up of such a report? If not, we will still report the adverse event, but will not provide the Sponsor with your contact details.

[If other IQVIA team members plan to listen in] We have a second IQVIA team member on what is called a listen-only line. While they will not be an active participant in our conversation, they are very interested in what you have to say. Do you give permission to have this second team member listen to the call?

Do you have any remaining questions about the study?

[IF YES, ANSWER QUESTIONS]

Do you agree to participate in the interview?

[IF YES, PROCEE BELOW]

Do you agree to have this interview audio-recorded?

[IF YES – PROCEED BELOW]

[IF NO] – The audio recording is a required part of the study as stated in the informed consent form. No identifying details will be shared outside of IQVIA unless required by law and all information shared will be anonymized so details cannot be traced back to you.

[IF AFTER EXPLANATION THE PARTICIPANT STILL DOES NOT WISH TO BE RECORDED] Unfortunately, the interview cannot proceed without audio recording. Thank you for the time. **[END CALL]**

[If no] – The audio recording is a required part of the study as stated in the informed consent form. No identifying details will be shared outside of IQVIA unless required by law and all information shared will be anonymized so details cannot be traced back to you.

[START RECORDING]

Can you reconfirm that you have agreed to be recorded and have consented to participate in the study?

Today is **[date]** and the time is **[time / moderator's time zone]**. This is interview ID **[participant / respondent ID provided by the SET or site]**.

2 BACKGROUND INFORMATION (5 MINUTES)

To begin, I would like to learn more about you and your background. For instance:

1. Can you confirm your age?
2. What do you do on a day-to-day basis? (e.g., work or school?)

3 CONCEPT ELICITATION OF EXPERIENCES WITH DISEASE AND TREATMENTS (20 MINUTES)

I'm going to ask you a few questions about your experience with HIV and your journey from diagnosis to where you are now.

First, let's start on how you normally describe your condition:

3. How do you refer to your condition when you mention it to others? Is there other term(s) you use? [Note: use the participant's preferred term for HIV for the remainder of the interview]

HIV DIAGNOSIS AND CARE

4. Please tell me, to the best of your memory, when were you officially diagnosed with [patient term for HIV – XX]? [Note: Take note of year of diagnosis as it may be used for sub-group analysis]

5. What type of healthcare professional (Doctor/Physician/ Nurse/ PAs) is involved with managing your [patient term for HIV – XX] now?
    a. Have you always had the same healthcare professional?
    b. How often do you currently visit this professional? Is this in the hospital, elsewhere, or both?
6. How would you describe your overall health status?

PREVIOUS INJECTION EXPERIENCE

7. Have you been diagnosed with any other diseases or medical conditions?
    a) If so, are your currently receiving treatment for them? **[If yes, probe questions below]**
        - If so, how long have you been receiving treatment for them?
        - Do you receive treatment in the form of pills, injections, or any other form?
    b) Have you ever received a treatment that involved continuous injections over an extended period for any disease? If yes, for which disease?

    [Note to moderator: Please clarify that this <u>does not include vaccinations</u> and that the previous injection experience should involve receiving injections at regular intervals, such as once a month or once every two months or longer, to deliver the medication. Typical examples of injectable treatments include diabetes, cholesterol, arthritis, etc.]

    If the PWH responds YES, ask 4.c to 4.e. If NO, skip to question 8]
    c) How long have you been receiving treatment through injections for this condition(s)?
    d) How frequently were these injections administered?
    e) Are these injections administered by a doctor or nurse, or are they self-injectable treatments?
    f) What reasons prompted you to switch to an injectable treatment for the <above mentioned disease>?
    g) Did you experience any side effects from these injections? What were they?

    [Pause to get a response. Then probe]
        - Redness, swelling, hardness or lumps, itching or rash at the injection site (injection site reactions)
        - Visible marks or nodules from injections (injection indentation)
        - Weight gain
        - Diabetes (metabolic issues)
        - Nausea, vomiting, diarrhea, constipation (gastric issues)
        - Other?

HIV TREATMENT EXPERIENCES

Now, I would like to know the treatments for HIV you have undergone so far.

8. What treatment(s) have you received for HIV since you were diagnosed with [patient term for HIV – XX] until today?

   [Note: please, list all treatments and ask the following questions per treatment type]

9. Can you please confirm the name of your current HIV treatment?

   Interviewer notes:
   - If PWH mentions “Cabenuva” or “Cabotegravir and Rilpivirine”, please proceed to Question 9i.
   - If PWH mentions treatments other than “Cabenuva” or “Cabotegravir and Rilpivirine”, please proceed to Question 10.

   i. Are you currently receiving “Cabenuva” or “Cabotegravir and Rilpivirine”? [If yes, continue to question iii – vii.

      If no, continue to question viii]

   ii. How do you feel about receiving Cabenuva injections once a month/every 2 months?

   iii. Would you feel comfortable self-administering Cabenuva injections using an autoinjector into the abdomen or thigh, or do you prefer having a healthcare professional administer them?

   iv. Are there any aspects of this treatment that you would change if you could?

   v. How would you describe your overall satisfaction with the Cabenuva treatment?

   vi. From your perspective, what are the biggest changes you’ve experienced taking Cabenuva compared to oral ART?

   vii. If given the opportunity, would you consider switching from Cabenuva to an alternative treatment?
      - What reasons would prompt you to make the switch?
      - On the other hand, what reasons would lead you to decide against switching from Cabenuva to an alternative treatment?

   viii. [Applicable only to PWH who have discontinued Cabenuva] Can you tell me why you stopped using Cabenuva / Cabenuba and Rilpivirine?
      - Did you switch to a different treatment? If so, could you tell me why you made that change?

10. Could you please tell me how did you administer each of these HIV treatments — through intravenous (IV), subcutaneous (SC), or oral administration?

[Interviewer notes]: If necessary, take some time to describe the different modes of administration to the participant by using the following definitions
Subcutaneously means that a shot is given under the skin using a small needle to inject the treatment into the tissue between the skin and muscle.
Intravenously means that an infusion is given into the vein for a period of time.
Orally means that you should take a pill (i.e., tablet or capsule).

FOR PWH TAKING ORAL MEDICATION:

i. How do you feel about taking your treatment at home?
ii. Does it offer any advantages or challenges compared to receiving treatment at a hospital or treatment center?
iii. Do you discuss your HIV treatment with others? If so, how do you feel about sharing this information with them?
iv. Do you take your medication in front of others or alone? What are the reasons for your choice?

FOR PWH TAKING IV AND/OR SC MEDICATION:

a. Where have you typically received these treatments? Have they been administered at home or in a hospital/treatment clinic/community center?
    i. Which of these locations would you prefer for receiving HIV treatments?

[Interviewer notes]:

- **If PWH answers "at the hospital, treatment or community center", then ask the following questions (i. to v.)**

    i. Who has been responsible for administering these treatments? Has it been done by a nurse or doctor?
    ii. How do you feel about taking your treatment at the hospital or treatment clinic/ community center?
    iii. How often do you visit the hospital or treatment clinic/ community center to receive treatment?
    iv. How long does it take you to travel from your home to the hospital or treatment clinic/ community center? Are there any challenging aspect or burdens associated with this travel?
    v. On average, how much time does your treatment appointment at the hospital or treatment clinic/ community center usually take?

11. How satisfied are you with your current medication to treat [patient term for HIV – XX]? Can you explain why?
12. What would you say is the most burdensome aspect related to each of these treatments? Why so?
13. Do you experience any other challenges with your current treatment than what's already been discussed? If so, can you please elaborate on these challenges?

14. In terms of your experience and what you know from discussions with your healthcare providers/other patients/online, what is missing in treatment for [patient term for HIV – XX] for you? [Pause to get a response, then probe]
    i. Convenient dosing schedules, e.g., fewer doses, longer lasting medications, etc.
    ii. Affordability, e.g., more affordable treatment options or assistance programs to improve access to treatments
    iii. Other?

HIV TREATMENT IMPACT

15. Now, I would like to understand the impact of the HIV treatments you have received on your overall well-being.
    a. How has your overall health and well-being been impacted by the [patient term for HIV – XX] treatments you have received?
    b. How easy or difficult has it been for you to adhere to your [patient term for HIV – XX] treatment regimen?
    c. Have you experienced any challenges in accessing or obtaining your [patient term for HIV – XX] medications? If so, could you describe them?
    d. Have you encountered any financial challenges related to the cost of [patient term for HIV – XX] medications or treatment? How have you managed them?

HIV TREATMENT SWITCH

[Moderator's note: If the reasons for switching treatments were previously covered in question 9 for patients using Cabenuva, **please skip questions 16 to 18**]

16. Have you ever had to switch or change your [patient term for HIV – XX] treatment regimen? [If yes, probe question below]
    a. What were the reasons behind that decision?
    b. Was this primarily yours or your clinician's decision?

17. If you were considering switching to another treatment that would be equally effective at treating your HIV, what would you say are the <u>most important</u> treatment characteristics that you would take into consideration when choosing whether or not to switch to a new treatment? [Pause to allow for a spontaneous answer, then probe]
    a. Why are these characteristics the most important?

[Note, Q18 is a low priority question, as we can get a more comprehensive answer in Q22]

18. Are there any characteristics that you consider to be <u>less important</u> when it comes to deciding on the best HIV treatment for you?
    a. Why these characteristics are less important to you?

4 SCREENSHARE SETUP (0.5 MINUTE)

[Interviewer script]

[Note to moderators: Screenshare should display slide 2 having the full list of attributes]

Next, I'd like to make sure you are connected to our screenshare platform. This screenshare platform will allow me to show you the list of attributes for the ranking exercise that we will be completing [please see example below].

List of Attributes for HIV Treatment*		
Mode of administration<br>*This is the way that a medication is delivered to the body.*	• Injection to a muscle (Intramuscular injection)<br>• Injection under the skin (Subcutaneous injection)<br>• Injection into a vein (Intravenous injection)<br>• A mixture of injection into the muscle and into a vein? (mixed-modality)<br>• A mixture of an initial infusion into a vein and an injection under the skin? (mixed-modality)<br>• Tablets or pills (Oral medication)	
Treatment administration setting<br>*This is the place where a medication is given.*	• HCP-administered injectables<br>• Self-administered injectables<br>• Administered at home (tablets or pills)	
Injection administration frequency<br>*How often an injection is given.*	• Once in 1 months (Q1M) (self-administered)<br>• Once in 2 months (Q2M) (self-administered)<br>• Once in 3 months (Q3M) (self-administered)<br>• Once in 4 months (Q4M) (HCP-administered)<br>• Once in 6 months (Q6M) (HCP-administered)	• Other? ________________
Late dose leeway<br>*How much time you can delay taking your medication before your viral load becomes detectable and the risk of drug resistance increases.*	For injection treatments:<br>• 2 weeks –for monthly or bi-monthly injection treatments<br>• 1 month –for treatments administered once every 3 months, or longer injection treatments<br>• 3 months –for treatments administered once every 4 months, once every 6 months, or longer injection treatments<br>• Other?	For weekly oral treatments<br>• 1 to 3 days<br>• Up to 1 week<br>• Up to 2 weeks<br>• More than 2 weeks<br>• Other?
The location of injection on body and number of injections<br>*The place on the body where an injection is given.*	• Thigh<br>• Glutes or Buttocks<br>• Abdomen<br>• Arm<br>• 1 injection<br>• 2 injections	• Other?________________
Side effects<br>*These are unwanted or unexpected effects that can occur after taking a medication. Side effects can vary from mild to severe, and they can be short-term or long-term.*	• Redness, swelling, hardness or lumps, itching or rash or pain at the injection site<br>• Discoloration or a change in skin appearance at the injection site, especially if you have darker skin<br>• Visible marks or nodules from injections	• Weight gain<br>• Diabetes<br>• Nausea, vomiting, diarrhea, constipation<br>• Other?________________
[Other characteristics…]*		

[Share screen to show the list of attributes to be discussed to facilitate the ease of participants]

5 DESCRIPTION OF TREATMENT ATTRIBUTES TO CAPTURE PREFERENCES (20 MINUTES)

19. Now, I will provide a list of treatment characteristics that may be relevant when considering a switch to an alternative treatment for HIV with the <u>same efficacy</u>. I would like you to share your thoughts on whether each characteristic is important or not when considering a new treatment. Please, feel free to elaborate on your response.

    [Interviewer notes: Please, explore each specific attribute covered in the table, one by one. Then take note of nay new or unprompted treatment attribute for the next ranking exercise.]

Questions/ Probes

Treatment Modality (Mode of administration)

i. Are you aware of the different ways HIV treatment can be administered to you?

[Interviewer notes] If necessary, take some time to describe the different modes of administration to the participant by using the following definitions: **Intramuscularly** means the shot is given directly into the muscle. **Subcutaneously** means that a shot is given under the skin using a small needle to inject the treatment into the tissue between the skin and muscle. **Intravenously** means that an infusion is given into the vein for a period of time. **Orally** means that you should take a pill (i.e., tablet or capsule)]

[Pause to get a response and then probe]

ii. If you were to switch HIV treatments, do you have a preference for how the treatments are administered?

[Pause to get a response. Then probe]

iii. Do you ever consider…?
   a. Injection to a muscle (Intramuscular injection)
   b. Injection under the skin (Subcutaneous injection)
   c. Injection into a vein (Intravenous injection)
   d. A mixture of injection into the muscle and into a vein? (mixed-modality)
   e. A mixture of an initial infusion into a vein and an injection under the skin (mixed-modality)
   f. Tablets or pills (Oral medication)

Oral Modality

i. If you were to keep an oral treatment, would you prefer to take daily oral medication or weekly oral medication? Can you please explain your choice?

Mixed Modality:

ii. If you were to switch treatments, would you prefer to take daily oral medication plus self-administered once monthly injections? Can you please explain your choice?

iii. When deciding on a treatment, what are the reasons that influence your decisions to switch treatments?

[Note to moderators: Please skip the factors listed below if they were already mentioned by the participant.]
[Pause to get a response. Then probe for factors missed by participants]

iv. Do you ever consider…?
   a. The schedule for taking the medication (e.g., how many doses, specific times to take it, instructions on how to take it)
   b. How often you need to take the medication (e.g., daily, once a week, once a month, twice a year)
   c. Side effects such as redness, swelling, hardness or lumps, itching or rash at the injection site (injection site reactions)
   d. Other?

Treatment Administration Setting

v. For treatments that include injections, would you rather give it yourself or have medical staff do it? Why so?

[If prefers HCP-administered injections → probe the following two sub questions, vi and vii; If prefers Self-administered injections → continue with questions viii and ix below]

vi. What factors would you consider when deciding to switch to a treatment administered by a medical professional?

[Pause to get a response. Then probe further]

vii. Are any of the following factors important to you when considering a switch to a treatment administered by a medical professional? Why so?
   a. Fear of needles or injections
   b. The location where the treatment is administered (e.g., home vs clinic vs pharmacy vs community hub)
   c. How long the treatment appointment takes
   d. How easy is to follow the treatment
   e. How easy is to schedule injection appointments
   f. Cost of the medication
   g. Availability of patient support services
   h. Other?

[If prefers self-administered injections, probe the following two sub questions]

viii. What factors would you consider when deciding to switch to an injection treatment delivered by yourself?

[Pause to get a response. Then probe]

ix. Do you ever consider…?
   a. Fear of needles or injections
   b. Features of the injection device (ease of use, convenience etc.)
   c. Cost of the medication
   d. Storage and disposal requirements
   e. Availability of patient support services
   f. Other?

[Note to moderator: Regardless of whether the subject prefers HCP or self-administered treatment, the following questions under both categories should be asked.]

Injection Administration Frequency

For HCP administered injectables

x. If you had the option to choose your treatment schedule, how frequently would you prefer to have a medical professional provide injections if you were to switch treatments?

[Pause to get a response and then probe]

xi. Which of the following injection frequencies are more relevant to you when deciding whether to switch treatments? Why so?
   a. Once in 4 months (Q4M)
   b. Once in 6 months (Q6M)
   c. Other?

xii. When thinking about a new treatment that includes injections, what things do you consider when thinking about how often you need to take them?

[Pause to get a response and then probe]

xiii. Do you ever consider…?
   a. Your lifestyle (e.g., your work or school commitments)
   b. Cost considerations, including medication and travel expenses
   c. Your relationship with healthcare professionals
   d. Side effects such as redness, swelling, hardness or lumps, itching or rash at the injection site (injection site reactions)

e. Other?

For self-injectables:

xiv. I have a list of frequencies for self-admin injectables, and I would like to know your opinion. Which of the following self-injection frequencies would you prefer if you were to switch treatments?

a. Once in 1 month
b. Once in 2 months
c. Once in 3 months
d. Other?

xv. Are there any factors that you consider when thinking about how often you need to self-inject the treatment?

[Pause to get a response and then probe]

xvi. Do you ever consider…?

a. Your lifestyle (e.g., your work or school commitments)
b. Cost of the medication
c. The features of the injection device
d. The storage needs for the treatment (e.g., whether you need to store it in a fridge)
e. The disposal of the injection device (e.g., whether you must follow specific disposal procedures or go to designated places for disposal)
f. Side effects such as redness, swelling, hardness or lumps, itching or rash at the injection site (injection site reactions)
g. Other?

Late dose leeway [forgivability]

I would like to ask you about a treatment feature known as **"late dose leeway".** This refers to how much time you can delay taking your medication before your viral load becomes detectable and the risk of drug resistance increases.

[Moderator, add if needed: This means that while keeping your viral load undetectable requires that you take your treatment as often as it is prescribed, some treatments allow for some flexibility in case you are late in taking your dose and still be OK.]

i. If you had the choice, what is the minimum amount of leeway for a late dose that would be acceptable for you if you were considering switching to injection treatments?

[Pause to get a response and then probe]

ii. Do each of the following late dose leeway options feel acceptable to you when deciding whether to switch to an injection treatment? Why so? [Moderator, go through the options and ask for each if is it acceptable]

a. 2 weeks –for monthly or bi-monthly injection treatments
b. 1 month –for treatments administered once every 3 months, or longer injection treatments
c. 3 months –for treatments administered once every 4 months, once every 6 months, or longer injection treatments
d. Other?

iii. And if you had the choice, what is the minimum amount of leeway for a late dose that would be acceptable for you if you were considering switching to a weekly oral treatment?

[Pause to get a response and then probe]

iv. Which of the following late dose leeway options feels more acceptable to you when deciding whether to switch to a weekly oral treatment? Why so?

a. 1 to 3 days
b. Up to 1 week
c. Up to 2 weeks
d. More than 2 weeks
e. Other?

The location of injection on body and Number of Injections

For HCP administered injectables

i. In which part of your body and what length of injection would you prefer if you were to switch to a treatment that was delivered by a medical professional? Why so?

[Pause to get a response and then probe]

ii. I have a list of possible locations. Which of the following locations would you prefer if you were to switch treatments? Why so?

a. Thigh
b. Glutes or Buttocks
c. Abdomen
d. Arm
e. Other?

iii. Now I have a list of possible locations and durations of the injections. Which of the following modalities would you prefer if you were to switch treatments?

a. Glutes or Buttocks – 2 consecutive injections within interval of 15mins, 20mins, 30 mins
b. Abdomen – 2 consecutive injections within interval of 15mins, 20mins, 30 mins
c. Arm – 2 consecutive injections within interval of 15mins, 20mins, 30 mins
d. Other?

iv. Is there anything about the location of the injection on the body that influences your choice to switch treatments when delivered by a medical professional?

[Pause to get a response and then probe]

v. Do you ever consider…?

a. Where it's most convenient to inject the medication on your body
b. Your relationship with healthcare professionals
c. Side effects such as redness, swelling, hardness or lumps, itching or rash at the injection site (injection site reactions)

For self-injectables:

vi. If you were going to administer the treatment yourself in the abdomen area, how much time would you prefer each injection to take if you were to switch to that treatment?
[Pause to get a response and then probe]

vii. I have a list of possible locations and durations of the injections:

a. Abdomen – 1 injection with a 20 sec duration
b. Abdomen – 2 consecutive injections with a 20 sec duration each
c. Other?

viii. Is there anything about the location of the injection on the body that influences your choice to switch treatments when delivered by yourself?
[Pause to get a response and then probe]

ix. Do you ever consider…?

a. Where it's most convenient to inject the medication on your body

b. How easy the device is to use
c. How private or discreet the injection can be (i.e., keeping it secret or maintaining privacy)
d. Any fear or anxiety you might have about needles
e. Side effects such as redness, swelling, hardness or lumps, itching or rash at the injection site (injection site reactions)

Number of injections [regardless of the location on the body and the administrator]

i. Now that you have considered different body locations and administrators, would you prefer 1 or 2 injections at each administration? Why so?

Side effects

i. If you were to switch treatment, what side effects or undesirable symptoms would you want to avoid? [Pause to get a response and then probe]

ii. Are there any of the following side effects or undesirable symptoms particularly concerning if you were to switch treatments?
   a. Redness, swelling, hardness or lumps, itching or rash or pain, discoloration at the injection site (injection site reactions)
   b. Discoloration or a change in skin appearance at the injection site, especially if you have darker skin (injection site reactions)
   c. Visible marks or nodules from injections (injection indentation)
   d. Weight gain
   e. Diabetes (metabolic issues)
   f. Nausea, vomiting, diarrhea, constipation (gastric issues)
   g. Other?

- Would you consider any other concept as important that we didn't cover that you would like to share? If so, what are they?

6 RANKING OF ATTRIBUTES (5 MINUTES)

[Note to Moderator: **Use your notes to collect and summarize the treatment-related features or attributes discussed thus far,** and then read them back to the participant, incorporating their words wherever possible. Please present this information using a PowerPoint slide deck or other appropriate method to share your screen. Once you have reviewed the attributes with the participant, proceed with the ranking exercise.**]**

[Interviewer script]

I have listed the characteristics of HIV treatments that we have discussed. Now, I would like to know which treatment factors or characteristics are most important to you when deciding to switch to future HIV treatments.

20. Would you please rank these attributes when deciding whether to switch or not switch to a future HIV treatment from most important to least important, where 1 indicates the most important and [5, 6, or total number of factors] indicates the least important?

21. Out of this list, could you please name the 3 attributes that would have the most impact on your decision whether to switch or not switch to a future HIV treatment?
    a. What leads you to see these attributes as the most important?

22. Conversely, could you please name the 3 attributes that have the least impact on your decision whether to switch or not switch to a future HIV treatment?

    b. What leads you to see these attributes as the least important?

7 TREATMENT SCENARIOS OR VIGNETTES (5 MINUTES)

[Share screen to show the hypothetical scenarios (vignettes) facilitate the ease of participant]

[Interviewer script] Next, I would like to show you the case of a hypothetical PWH who we will call <vignette’s name> who is undergoing treatment. <vignette’s name> is being offered different treatment options with varying characteristics. We are going to use a think-aloud method. With the think-aloud method, you will read all content that you see in the vignette (including the instructions, items, and answer choices) and verbally tell me your answer choice to each item and why. I will also be asking you some questions about your preferences as you go through the vignette, and I may occasionally remind you to think-aloud. [Present the vignette via the screenshare platform] Please begin reading aloud.

Instructions	*Show vignette(s)*

23. Can you please read <vignette’s name> out loud?
24. Can you please describe in your own words what this patient profile is describing? [Moderator, we are not directly asking if understandable, instead, our team will assess based on the respondent’s recap of what the vignette say whether they ‘understood’ the intent)
25. Was this vignette clear as you read it?
    a. Were there any elements that were confusing?
    b. Does any of the information in this profile stand out to you or seem unusual / out of place?
    c. What would you change in the wording to make it clearer? What may make it clearer?

[Moderator to ask these following questions at the end of all the vignettes or scenarios].

26. Now, I would like to know your opinion regarding the mode of administration displayed in this treatment switch scenario:
    d. Which mode of administration are you most comfortable with?
    e. Which mode of administration would you like to be sure to avoid?
27. Similarly, I would like to know your opinion regarding the frequency of administration displayed in this treatment switch scenario:
    f. What frequency of administration are you most comfortable with?

  g. What frequency of administration would you like to be sure to avoid?

28. Imagine that you were Mia, are there any treatment or patient characteristics described in this switching scenario that you consider important if you were to switch treatment? Why or why not?

8 WRAP-UP (2.5 MINUTES)

These are all of the questions that I have for you on this topic. Your answers have been extremely insightful. Do you have any other comments or experiences that you would like to share?

If additional information is required to complete adverse event reporting, ask for additional detail here

Thank you for your time. As a token of our appreciation, we will be compensating you. Please allow four to six weeks to receive an email notice of your compensation.

Today is **[date]** and the time is **[time / moderator's time zone]**. This is interview ID **[participant / respondent ID provided by the SET or site]**.

[END RECORDING]

9 APPENDIX: VIGNETTE

Vignette 1: Mia's Story (Transitioning from monthly self-injections to HCP-administered injections into the arm muscles, on a 4-monthly basis)

Mia, a 28-year-old woman living with HIV has recently become homeless. Mia has transitioned from monthly self-injections to receiving injections given by her healthcare provider in her arm muscles every 4 months. This change in her treatment means Mia does not to need to find refrigeration storage for her medication and reduces the need for frequent injections and healthcare visits. However, she does have concerns about the visible nodule marks [or bumps] that appear in her upper arm after the injection. Mia plans to consult with her clinician to address these concerns and seek further guidance. Additionally, she receives support from a social worker who provides timely reminders for her injection appointments, ensuring that Mia stays on track with her treatment schedule.

Scenario – During the interview, participant reports active thoughts of harm (this includes suicidal ideation as well as any physical injury to themselves or others):

Moderator*: "You described having thoughts of hurting yourself. Although I am not a trained clinician to help you deal with those thoughts directly, I do have a responsibility to ensure as much as possible over the phone that you are safe. Can you please let me know if you are actively (past 24 hours) thinking about hurting yourself or someone around you?"*

- If the participant says yes: *"I want to remind you that during the consent process, you consented to have your doctor and / or emergency services personnel contacted in case of emergency. As this is considered an emergency, I will be contacting [insert third-party as appropriate] immediately. I want to ask that you stay on the phone with me as I try and connect us both with one of my supervisors who is better trained to help people when they are feeling like they want to hurt themselves or others / feel there is no hope."* **[Keep your voice compassionate, but firm]**

  The moderator needs to decide who to contact (designated senior scientist, or the project PIC / EL). If no one is available to be reached on the phone, it is up to the moderator to decide, with a reasonable degree of certainty, if the participant is safe to end the call. Prior to ending the call, provide them with the suicide hotline (**National Suicide Prevention Line - https://suicidepreventionlifeline.org/; Tel: 1-800-273-8255**). Also encourage them to consider immediately calling 911 (or their country's local equivalent) or immediately go to the local emergency room.

  If the moderator feels that the participant is in imminent danger, stay on the phone with them and continue engaging in a discussion with them. Continue to assure them that you will stay on the phone until you are able to get in contact with someone that is trained to assist. Try to get in contact once more with members of the team to help. If not successful, call 911 (or their country's local equivalent).

- If the thoughts are not active: Contact the participant's doctor and / or emergency services personnel, if needed / as appropriate, and provide them with the suicide hotline (**National Suicide Prevention Line - https://suicidepreventionlifeline.org/; Tel: 1-800-273-8255**).
