# Supplementary material for: Preferences of people living with HIV for treatment regimen attributes: a qualitative interview study
Source: Front Public Health. 2026 Sep 14;14:1907747. doi: 10.3389/fpubh.2026.1907747 (PMC13617073; doi:10.3389/fpubh.2026.1907747)
Supplement: Supplementary file 2 [file Data_Sheet_2.pdf]

Supplementary File S2. Patient Preferences for Regimen Attributes for Treatment of HIV, August 2023.

Preferences For Regimen Attributes For Treatment Of HIV

-- HCP Discussion Guide –

August 3rd, 2023

DOCUMENT'S SPECIFICATIONS

**TITLE**	**Preferences For Regimen Attributes For Treatment Of HIV – HCP Discussion Guide**
**VERSION**	**V.03**
**DATE**	**August 3rd 2023**
**CONDUCTED BY**	**IQVIA**

DOCUMENT'S PURPOSE

This discussion guide has been designed to conduct semi-structured interviews with HCPs who specialize in treating persons with HIV (PWH) and who have experience prescribing or administering HIV treatment, particularly long-acting injectable therapy and daily/weekly oral ART. **The nature of the interview will provide insights related to related to clinician preferences for long-acting injectable and oral antiretroviral therapy (ART) compared to daily oral therapy, and to compare different long-acting injectable options influencing clinician preferences.** The specific objectives of these interviews are to evaluate clinician preferences and identify the key attributes associated with short or longer acting injection options. Additionally, these interviews aim to gather HCPs' perspectives on the experiences of PWH in managing and administering treatments, and to describe existing gaps in the treatment options available to PWH.

INTERVIEW STRUCTURE AND TIMING

**Interview with HCPs treating/managing HIV**		
**Guide section**	**Objective**	**Timing (min)**
1. Introduction and disclosures	Explain the purpose of the interview, obtain informed consent, and disclose relevant study information.	2
2. Background information	Gather clinical experience information about the participant.	5
3. Concept elicitation of experiences with HIV and treatments	Understand the participant's clinical experiences with HIV, including treatments and view on PWH.	15
4. Screenshare set-up	Facilitate a visual presentation of attributes and ranking.	1
5. Description of treatment attributes to capture preferences	Capture the participant's thoughts, and preferences regarding each treatment attribute.	15
6. Ranking of attributes	Engage the participant in ranking the importance of treatment attributes based on their preferences.	5
7. Treatment scenarios or vignettes	Facilitate a deeper exploration of factors that influence HCP’s perspectives on ART-related risks and benefits.	15
8. Wrap-up	Provide the participant with an opportunity to share any additional insights.	2
	**Total**	**60**

1 INTRODUCTION AND DISCLOSURES (2 MINUTES)

[Note to reader – the text here is intended to be a “guide” and not to be read verbatim. An experienced moderator will plan to cover these topics but may use alternative language depending on the natural direction of the conversation.]

Hello. This is **[Moderator Name]** from IQVIA for our scheduled interview. This interview will take approximately 60 minutes. Is this still a good time to talk?

[If no] – Is there another time that works for you? **[Reschedule as appropriate]**

[If yes – proceed below]

As a reminder, the purpose of this 60-minute interview study is to speak with HCPs, as yourself, who have experience in treating persons with HIV (PWH) to understand the treatment preferences associated with long-acting therapies and daily/ weekly ART (antiretroviral therapy). The interview will be divided into two sections

1. In the first section, we will be asking about your thoughts on certain aspects of HIV treatment. In addition, we will explore your opinions towards key treatment characteristics, how important these characteristics are to you and the trade-offs between these characteristics.
2. In the second section, we will ask you to rank and rate treatment characteristics depending on your preferences. Next, we present you with some hypothetical scenarios related to HIV treatments and will ask your feedback related to how well you understand each scenario, what characteristics are missing and how it can be improved

We may discuss some sensitive topics. We will respect your privacy and if you feel uncomfortable answering a question, please let me know.

The plan is to audio-record this conversation today so that I am able to focus on our conversation and create a transcript of the interview after. Confidentiality is very important to us, so once we start recording, please do not use your name or the names of family, friends, or any patients you are treating. If you mention a name accidentally during the interview, we will redact it from the transcript to preserve your anonymity. Additionally, if we are connecting with you through a conferencing platform on a computer or mobile device, kindly ensure that your webcam remains disabled throughout the duration of the interview.

Any reports that result from these interviews will include data where all personally identifying information has been removed and / or reported at a combined level, which means that it will not be possible for you to be individually identified.

If you report an adverse event (for example, a side effect of a medication) related to any of the study Sponsor’s products, IQVIA is obligated to report this. If that occurs, are you willing to have your name and contact information sent to the Sponsor <and the site principal investigator> so that it can be passed on to the Sponsor’s drug safety department for the express and sole purpose of follow-up of such a report? If not, we will still report the adverse event, but will not provide the Sponsor with your contact details.

[If other IQVIA team members plan to listen in] We have a second IQVIA team member on what is called a listen-only line. While they will not be an active participant in our conversation, they are very interested in what you have to say. Do you give permission to have this second team member listen to the call?

Do you have any remaining questions about the study?

May we audio-record the interview?

[START RECORDING]

Can you reconfirm that you have agreed to be recorded and have consented to participate in the study?

Today is [date] and the time is [time / moderator's time zone]. This is interview ID [participant / respondent ID provided by the SET or site].

2 BACKGROUND INFORMATION (5 MINUTES)

To start, could you please share some background information about yourself and the clinical settings in which you provide treatment to PWH. For instance:

Could you confirm what is your specialty?

1. How many years of experience do you have in providing treatment and long-term follow-up care for people living with HIV (PWH)?
2. Where do you typically see individuals for the treatment and management of their HIV? (Community clinics / academic centers / public hospital / private clinic, etc.)?
3. Could you please describe the characteristics of the patient population with HIV that you encounter? [Probe on: African-American population, Hispanic/Latino population, gay, bisexual and MSM, individuals experiencing homelessness, individuals with history of drug addiction, other disadvantaged or vulnerable communities]
    - What are the primary challenges you face when working with each of these populations?
    - How do you typically address these challenges?

3 CONCEPT ELICITATION OF EXPERIENCES WITH DISEASE AND TREATMENTS (15 MINUTES)

[Please, adapt the wording when needed to apply for nurses and PA. For instance, instead of "prescribing", use "administering" or "assisting"].

Now, I would like to explore your perspectives on HIV treatments and understand the key treatment characteristics that could influence your choice when making treatment decisions for your patients. I would like to understand your perspective on the current

and future HIV treatment landscape and identify any gaps that you foresee in the current approach.

[Remember to take note in the stimuli slide of any new or prompted attribute for the ranking exercise.]

4. Let's start with your experience treating HIV. Do you have experience prescribing oral ARTs (antiretroviral therapy) to PWH? If so, could you describe it?
    a. Have you encountered any barriers or challenges in prescribing oral therapy in your practice?
    b. If so, what are they, and how do you address them?
    c. How satisfied are you with the current medications used to treat HIV? Can you explain why?
    d. In your experience, what gaps exist in the current range of treatments available to PWH?
    e. Are there any specific areas where more options or alternatives are needed?
5. Do you have experience prescribing long-acting injectable ARTs to PWH? If so, could you describe it?
    a. Have you encountered any limitations or concerns with long-acting injectable therapy that have influenced your treatment decision-making process?
        i. If so, could you please elaborate on them?
    b. Have you encountered any barriers or challenges in prescribing or administering long-acting injectable therapy in your practice?
        i. If so, what are they, and how do you address them?
    c. What are some of the specific barriers or challenges that you either currently experience or foresee in rolling out long-acting injectable ART in your clinic/organization? [Pause to allow for a spontaneous answer, then probe]
        i. Do you have the required trained staff to administer LAIs?
        ii. From your perspective, does insurance coverage affect adoption of LAI?
        iii. From your perspective, is accessing LAI medication difficult?
        iv. Is physical infrastructure and storage a barrier to LAI adoption?
        v. Does LAI adoption interfere with clinic workflow?
        vi. Do you experience or foresee increased paperwork associated with LAI?
            - [If so, probe] Does this increased paperwork primarily affect the patient or the clinic? Why so?
6. Do you have any specific expectations regarding long-acting injectable treatment options? [Pause to allow for a spontaneous answer, then probe]
    a. How do you perceive the convenience of long-acting injectable treatment options for people living with HIV?
        i. Are there any specific factors that you believe contribute to their convenience or lack thereof?
    b. What potential impact do you foresee long-acting injectable treatment options having on the lives of people living with HIV?

7. Are there any specific characteristics that make certain patient populations particularly well-suited for long-acting injectable ART?
    a. Conversely, are there any patient populations that you believe may not be suitable candidates for long-acting injectable therapy?
    b. What types of patient-related barriers do you think there are to using long-acting injectable ART?
    c. How do these challenges differ from those encountered with daily oral therapy?
    c. What factors might help or facilitate persons with HIV engage in long-acting injectable ART?

[Question 8 and 9 are only applicable for Physicians. Please proceed to section 4 for Nurses and PA's]

8. Now I would like to understand how you compare different ART modalities. Would you recommend a PWH to receive long-acting injectables over oral ART to treat HIV? Please explain why or why not.
    a. Are there any trade-offs or challenges that need to be considered when evaluating different long-acting treatment options?
    b. Have you ever switched a PWH treatment regimen from oral to long-acting injectable ART? Please explain why or why not.
    c. Conversely, have you ever switched a PWH treatment regimen from long-acting injectable to oral ART? Please explain why or why not.

9. The following questions will focus on your perspective of the future HIV treatment landscape, and in particular, the use of LAIs for ART.
    a. Do you envision LAI becoming a more common treatment request from PWH in the next 3 to 5 years? Why or why not?
    b. Do you foresee LAIs addressing the current gaps in the HIV treatment paradigm?
    c. Do you foresee any challenges or limitations for HCPs with increased prescriptions of LAIs?

4 SCREENSHARE SETUP (1 MINUTE)

[Interviewer script]

Next, I'd like to make sure you are connected to our screenshare platform. This screenshare platform will allow me to show you the list of attributes for the ranking exercise that we will be completing [please see example below].

List of Attributes

To be discussed with participants

Attributes for HIV Treatment
Mode of administration
Treatment administration setting
Treatment administration frequency
The location of injection on body
Side effects
[Other attribute…]*
[Other attribute…]*

**Note to moderators: Please edit attributes accordingly based on participant responses*

[Share screen to show the list of prompted and unprompted attributes as discussed above to facilitate the ease of participants]

5 DESCRIPTION OF TREATMENT ATTRIBUTES TO CAPTURE PREFERENCES (15 MINUTES)

Now, I would like to understand your personal satisfaction with the current treatments for HIV and explore the treatment attributes or characteristics that would lead you to consider switching to alternative treatment options.

10. How satisfied are you with the current medications used to treat HIV? Can you explain why?

11. In your opinion, what factors would help you to be more satisfied with a treatment for HIV?
    a. Are there any specific treatment attributes or characteristics that you feel are missing or could be improved in the current HIV treatments?
    b. What would prompt you to consider switching to an alternative treatment option that offers equivalent efficacy?

12. Now, I will provide a list of treatment characteristics that may be relevant when considering a switch to an alternative treatment for HIV with equivalent efficacy. I would like to hear your thoughts on the importance of each characteristic when considering a switch. Please, feel free to elaborate on your response.

[Interviewer notes: **Please, take note of any new or unprompted treatment attribute for the ranking exercise.** Note that any **treatment attributes mentioned spontaneously by the participant should be discussed first**. Afterward, **explore each specific attribute covered in the list**, one by one, **including any new additions**.]

When considering a switch to an alternative treatment with equivalent efficacy:

Questions/ Probes*

Mode of administration

i. To what extent is the mode of administration (MOA) relevant to your decision to switch treatments?
[Pause to get a response and then probe]

ii. Are any of the following characteristics of the MOA relevant to your decision to switch treatments?
   a. Intramuscular injection
   b. Subcutaneous injection
   c. Intravenous injection
   d. Mixed modality (e.g., a mix of oral medication and injections)
   e. Oral

   [Probe for each one identified as relevant]

iii. For which patient type is the mode of administration more relevant?

Oral Modality

iv. Would you prefer to prescribe a daily oral medication or prescribe a weekly oral medication? Can you please explain your choice?

v. When considering a treatment switch like this, what factors do you consider?
[Pause to get a response and then probe]
   a. Treatment schedule (e.g., how many doses, specific times to take it, intake instructions)
   b. Treatment frequency
   c. Side effects
   d. Other?

Mixed Modality

vi. Would you prefer to prescribe mixed modalities such as a daily oral medication and self-administered once monthly injections? Can you please explain your choice?

vii. When considering a treatment switch like this, what factors do you consider?
[Pause to get a response and then probe]
   a. Treatment schedule (e.g., how many doses, specific times to take it, intake instructions)
   b. Treatment frequency
   c. Side effects
   d. Other?

Treatment Administration Setting

<u>HCP-administered injectables</u>

viii. For treatments that include injections, would you prefer to prescribe a self-injectable or a HCP-administered injection?

ix. **[If prefers HCP-administered injections, probe]** When making the decision to switch to a HCP administered injectable treatment, what aspects do you typically consider?

[Pause to get a response and then probe further]

x. Do you ever consider…?
   a. The duration or length of time of a PWH appointment
   b. The time taken to administer the injection
   c. The scheduling of injection appointments
   d. The location where the treatment is administered (e.g., PWH's home vs clinic vs pharmacy vs community hub)
   e. The need for additional staff to administer the injections
   f. The requirement for a new equipment to administer the injections
   g. The need for treatment storage
   h. The need for refrigeration
   i. The availability of Patient Support Services
   j. Other?

[Probe for each one identified as relevant]

xi. For which patient type are these factors more relevant?

<u>Self-administered injectables</u>

xii. **[If prefers self-administered injections, probe]** When making the decision to switch to a self-administered injectable treatment, what aspects do you typically consider?

[Pause to get a response and then probe further]

xiii. Do you ever consider…?
   a. The patient's experience with self-injectables?
   b. The self-injection device (ease of use?)
   c. Cost of the medication
   d. Storage and disposal requirements
   e. Other

Treatment Administration Frequency

<u>For HCP administered injectables</u>

xiv. In your perspective, for HCP administered injectables, which frequency of injection would be more suitable?

[Pause to get a response and then probe]

xv. Which of the following injection frequencies are more relevant to you when deciding whether to switch treatments? Why so?
   a. Once in 4 months (Q4M)
   b. Once in 6 months (Q6M)
   c. Other?

[Probe for each one identified as relevant]

xvi. For which patient type is this frequency more relevant?

<u>Self-admin injectables</u>

xvii. For self-administered injectables, which <u>injection frequency</u> is more relevant to your decision to switch treatments?

[Pause to get a response and then probe]

xviii. From your perspective, which of the following self-admin injection frequencies are more relevant to your decision to switch treatments?
  a. Once in 1 months (Q1M)
  b. Once in 2 months (Q2M)
  c. Once in 3 months (Q3M)
  d. Other?

[Probe for each one identified as relevant]

xix. For which patient type is this more relevant?

The location of injection on body

For HCP administered injectables

xx. Do you have any preference regarding the location of injection and injection length when deciding to switch treatments? Why so?

[Pause to get a response and then probe]

xxi. I have a list of possible locations and injection length. Which location of injection on the body and length of injection is more relevant to your decision to switch treatments?
  a. Glutes or Buttocks – 2 consecutive injections within time frame of 15mins, 20mins, 30 mins
  b. Abdomen - 2 consecutive injections within time frame of 15mins, 20mins, 30 mins
  c. Arm – 2 consecutive injections within time frame of 15mins, 20mins, 30 mins
  d. Other?

[Probe for each one identified as relevant]

xxii. For which patient type is this more relevant?

For self-administered injectables:

xxiii. From your experience, which duration of injection is considered preferable for self-administered injectables in the abdomen area?

[Pause to get a response and then probe]
  a. Abdomen – 1 injection with a 20 sec duration
  b. Abdomen – 2 consecutive injections with a 20 sec duration each
  c. Other?

xxiv. In your experience, are there any other preferred locations of injection mentioned by patients, in addition to the abdomen?

[Probe for each one identified as relevant]

xxv. For which patient type is this more relevant?

Side effects

xxvi. What side effects/undesirable symptoms while taking treatments for HIV would you want to ensure the PWH avoids?

[Pause to get a response and then probe]

xxvii. Are there any of the following side effects or undesirable symptoms relevant to your decision to switch treatments?
  a. Injection site reactions (e.g., redness, swelling, hardness or lumps, itching or rash, pain, duration of pain, discoloration)
  b. Visible marks from injections
  c. Nodule indentations
  d. Weight gain
  e. Metabolic issues (e.g., diabetes)

f. Gastric issues (e.g., nausea, vomiting, diarrhea, constipation)<br>g. Other?<br><br>[Probe for each one identified as relevant]<br>xxviii. For which patient type is this side effect more relevant?
▪ Would you consider any other concept as important that we didn't cover that you would like to share? If so, what are they?

6 RANKING OF ATTRIBUTES (5 MINUTES)

[Interviewer script]

[Note to Moderator: **Use your notes to collect and summarize the prompted and unprompted treatment-related features discussed thus far,** and then read them back to the participant, incorporating their words wherever possible. Please present this information using a PowerPoint slide deck or other appropriate method to share your screen. Once you have reviewed the attributes with the participant, proceed with the ranking exercise.]

I have listed the HIV treatment characteristics that we have discussed so far. Now, I would like to understand which treatment characteristics or factors you would prioritize when making a decision to switch a patient with HIV to future treatments.

13. Out of this list, could you please name the main factors that would weight the most in your decision whether to switch or not switch to a future HIV treatment?

14. Would you please rank these factors that you just mentioned when deciding whether to switch or not switch to a future HIV treatment from most important to least important, where 1 indicates the most important and [5, 6, or total number of factors] indicates the least important?

    a. What makes <factor ranked first> most important?

    b. What makes <factor ranked last> least important?

15. Conversely, could you please name the factors that would carry the least weight in your decision whether to switch or not switch to a future HIV treatment?

7 TREATMENT SCENARIOS OR VIGNETTES (15 MINUTES)

[Share screen to show the hypothetical scenarios (vignettes) facilitate the ease of participant]

[Interviewer script]

Next, I would like to show you different treatment switching scenarios among hypothetical PWH. We are going to use a think-aloud method. With the think-aloud method, you will read all content that you see in the vignette, and I will also be asking you some questions about your

understanding as you go through the vignette, and I may occasionally remind you to think-aloud. [Present the vignettes via the screenshare platform] Please begin reading aloud.

Instructions	Show vignette(s)

16. Can you please read <vignette's name> out loud?
17. Can you please describe in your own words what this patient profile is describing? [Moderator, we are not directly asking if understandable, instead, our team will assess based on the respondent's recap of what the vignette say whether they 'understood' the intent]
18. Was this vignette clear as you read it?
    a. Were there any elements that were confusing?
    b. Does any of the information in this profile stand out to you or seem unusual / out of place?
    c. What would you change in the wording to make it clearer? What may make it clearer?
    d. Is there any of the details described in the vignette relevant to you? Why or why not?
19. Now, I would like to know your opinion regarding the mode of administration displayed in these treatment switch scenarios:
    a. What characteristics of the mode of administration are you most comfortable with?
    b. What characteristics of the mode of administration would you like to be sure to avoid?
20. Similarly, I would like to know your opinion regarding the frequency of administration displayed in these treatment switch scenarios:
    a. What frequency of administration are you most comfortable with?
    b. What frequency of administration would you like to be sure to avoid?
21. Is there any other treatment or patient characteristics described in these switching scenarios that you find relevant when considering a treatment switch for a similar patient? Why or why not?

8 WRAP-UP (2 MINUTES)

These are all the questions that I have for you on this topic. Your answers have been extremely insightful. Do you have any other comments or experiences that you would like to share?

Thank you for your time. As a token of our appreciation, we will be compensating you. Please allow four to six weeks to receive an email notice of your compensation.

Today is [date] and the time is [time / moderator's time zone]. This is interview ID [participant / respondent ID provided by the SET or site].

[END RECORDING]

9 APPENDIX: VIGNETTES

Vignette 1: Carlos's Story (Transitioning from weekly oral tablets to self-administered subcutaneous auto-injectors on a monthly basis)
Carlos, a 42-year-old man living with HIV, has transitioned from taking weekly oral tablets to self-administering subcutaneous injections using an autoinjector for his HIV treatment. Carlos now administers two consecutive injections in his abdomen every month. This change enables Carlos to administer his injections at home. With training from his healthcare provider on the injection technique, Carlos can now independently self-administer his HIV treatment. However, Carlos does have concerns about properly storing his medication at home, given it needs to be stored in the fridge. Carlos plans to discuss proper medication storage with his healthcare provider.

Vignette 2: Anna's Story (Transitioning from daily oral tablets to multiple ultra-long acting HCP-administered intramuscular injections on a 4-monthly basis)
Anna, a 65-year-old transgender woman living with HIV, has recently made the decision to transition from taking daily oral medication to receiving intramuscular injections administered by her healthcare provider in the gluteal (buttocks) area every 4 months. Anna no longer needs to remember to take medication daily. She appreciates the expertise of her healthcare provider in administering the injections.

Vignette 3: Mark's Story (Transitioning from daily oral tablets to HCP-Administered to mixed modality subcutaneous and IV Infusion on a 6-month basis)
Mark, a 49-year-old man living with HIV, has recently made the decision to transition from taking daily oral medication to receiving two 1-hour subcutaneous infusions into the abdomen and 15-minute IV infusion into the arm, administered by his healthcare provider every 6 months. This change in his treatment routine prevents Mark of needing to remember to take medication daily. However, he does have to plan for a day off to go to the clinic for the infusions.

Vignette 4: Mia's Story (Transitioning from self-administered monthly injections to HCP-administered intramuscular injections on a 4-monthly basis)
Mia is a 28-year-old woman living with HIV and has recently become homeless. Mia has transitioned from self-administering monthly injections to receiving intramuscular injections administered by her healthcare provider in the arm every 4 months. This change in her treatment allows her not to need to find storage for her medication and reducing the need for frequent injections and healthcare visits. However, she does have concerns about the visible nodules that appear in her upper arm after the injection. Mia plans to consult with her clinician to address these concerns and seek further guidance. Additionally, she receives support from a social worker who provides timely reminders for her injection appointments, ensuring that Mia stays on track with her treatment schedule.

Vignette 5: Sarah's Story (Transitioning from oral weekly tablets to ultra-long-acting HCP-administered subcutaneous and IV infusion on a 6-monthly basis)
Sarah, is a 65-year-old woman living with HIV who is also receiving treatment for diabetes and hypertension. Sarah has recently transitioned from taking oral medication once a week to receiving two ultra-long-acting 1-hour subcutaneous (SC) infusion into the abdomen, and 15-minute IV infusions into the arm administered by her healthcare provider every 6 months for her HIV treatment. This treatment approach offers Sarah the advantage of longer intervals between doses, reducing the frequency of medication administration, and allows her to focus on taking her daily insulin injections and statin pills. With this transition, Sarah feels more in control of her

HIV treatment as she can rely on the expertise of her healthcare provider for the administration of these injections.
